# DYRK1B phosphorylates FOXO1 to promote hepatic gluconeogenesis

**DOI:** 10.1093/nar/gkaf319

**Published:** 2025-04-26

**Authors:** Shanshan Li, Kai Huang, Chu Xu, Hong Zhang, Xiao Wang, Rong Zhang, Yan Lu, Man Mohan, Cheng Hu

**Affiliations:** Shanghai Diabetes Institute, Shanghai Sixth People’s Hospital Affiliated to Shanghai Jiao Tong University School of Medicine, Shanghai 200233, China; Department of Sports Medicine, Shanghai Sixth People’s Hospital Affiliated to Shanghai Jiao Tong University School of Medicine, Shanghai 200233, China; CAS Key Laboratory of Genome Sciences and Information, Beijing Institute of Genomics, Chinese Academy of Sciences, Beijing 100101, China; Shanghai Diabetes Institute, Shanghai Sixth People’s Hospital Affiliated to Shanghai Jiao Tong University School of Medicine, Shanghai 200233, China; Key Laboratory of Biomedical Research Center, Sir Run Run Shaw Hospital, Zhejiang University School of Medicine, Hangzhou 310002, Zhejiang, China; Shanghai Diabetes Institute, Shanghai Sixth People’s Hospital Affiliated to Shanghai Jiao Tong University School of Medicine, Shanghai 200233, China; Institute of Metabolism and Regenerative Medicine, Shanghai Sixth People’s Hospital Affiliated to Shanghai Jiao Tong University School of Medicine, Shanghai 200233, China; State Key Laboratory of Primate Biomedical Research, Institute of Primate Translational Medicine, Kunming University of Science and Technology, Kunming 650500, China; Shanghai Diabetes Institute, Shanghai Sixth People’s Hospital Affiliated to Shanghai Jiao Tong University School of Medicine, Shanghai 200233, China

## Abstract

Dual-specificity tyrosine phosphorylation-regulated kinase 1B (DYRK1B), a member of the CMGC group of kinases, is linked to metabolic syndrome, though the underlying molecular mechanisms remain unclear. In this study, we show that *Dyrk1b* expression is induced in the liver by fasting and in diabetic mice. Through both *in vivo* and *in vitro* experiments, we demonstrate that DYRK1B promotes hepatic gluconeogenesis and glucose intolerance. Liver-specific *Dyrk1b* conditional knockout mice were protected from diet-induced hyperglycemia. Mechanistically, DYRK1B interacts with and phosphorylates FOXO1, primarily at Thr467/Ser468, which is essential for its nuclear localization. Additionally, DYRK1B inhibits AKT-mediated FOXO1 phosphorylation at Thr24 and Ser256, enhancing its nuclear retention. DYRK1B-mediated phosphorylation increases the expression of gluconeogenic genes and promotes gluconeogenesis. Further, AZ191, a pharmacological inhibitor of DYRK1B, significantly reduced blood glucose levels in diabetic mice. Collectively, these findings provide new insights into the role of DYRK1B in glucose metabolism and identify it as a new therapeutic target for treating diabetes.

## Graphical Abstract

**Graphical Abstract:**
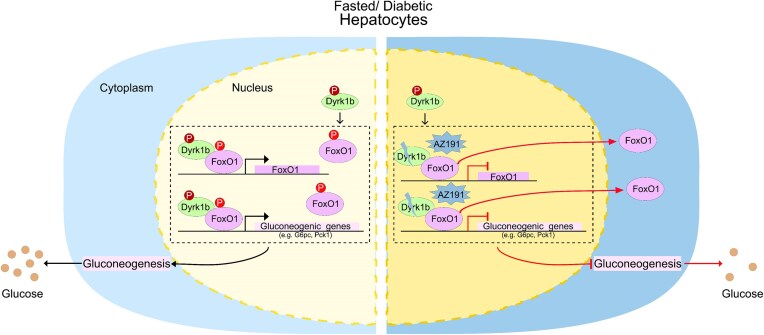

## Introduction

Type 2 diabetes mellitus (T2DM) is a metabolic disease characterized by elevated circulating glucose levels, usually accompanied by insulin resistance in liver, skeletal muscle, and adipose tissue [1, 2]. Insulin resistance results in decreased glucose uptake in skeletal muscle and adipose tissue, elevated glucose production in liver, and subsequent hyperglycemia [2, 3]. In the liver, glucose is synthesized from noncarbohydrate substrates, such as amino acids or lactate. This biochemical process is known as gluconeogenesis and hepatic glucose production (HGP) [4–6]. Prolonged fasting or starvation requires glucagon to activate gluconeogenesis, thus ensuring energy supply to the brain and survival of the individual [1, 3, 7]. Conversely, feeding requires insulin to inhibit hepatic gluconeogenesis and HGP to ensure glucose homeostasis [3, 8, 9]. The inability to inhibit hepatic gluconeogenesis due to insulin resistance is the main cause of hyperglycemia in patients with T2DM [1, 5, 6]. Therefore, suppressing the hyperactivation of hepatic gluconeogenesis has been considered an effective pharmacological intervention for treating T2DM.

The regulation of hepatic gluconeogenesis primarily occurs at the transcriptional level of rate-limiting enzymes, including glucose-6-phosphatase (G6pc, G6Pase) and phosphoenolpyruvate carboxykinase (Pck1, PEPCK), by a set of transcription factors and coactivators, including FOXOs, CREB, HNF4α, CRTC2, and PGC-1α [3, 5, 6, 10]. CREB and CREB-regulated transcription coactivator 2 (CRTC2) and PCG1 upregulate gluconeogenesis in response to glucagon, while forkhead O proteins (mainly FoxO1 and FoxO3) downregulate the process under insulin stimulation [3, 6, 10–12]. FoxO1, central to energy metabolism, is phosphorylated by protein kinase B (also known as Akt) upon insulin or growth factor stimulation, leading to its cytoplasmic translocation and repression of target gene expression [13–16].

Dual-specificity tyrosine phosphorylation-regulated kinase 1B (DYRK1B) is a highly conserved protein kinase of CMGC group of the eukaryotic kinases [17]. DYRK1B undergoes autophosphorylation on its tyrosine residues (Tyr-271/273) within the kinase domain for activation [18, 19]. Various studies have revealed that DYRK1B plays multiple roles in both physiological and pathological processes [18, 20–22], and genome stability [23, 24]. Interestingly, a mutation in DYRK1B at arginine residue (R102) is associated with metabolic syndrome and cardiovascular disease [20]. Since then, other reports have linked mutations in DYRK1B to monogenic obesity [25] and abdominal obesity-metabolic syndrome 3 [26]. Recent studies have suggested mechanisms by which DYRK1B functions in cardiac dysfunction [21], hepatic lipogenesis [22], and enteroendocrine system [27]. However, the involvement of DYRK1B in hepatic gluconeogenesis and HGP has not yet been investigated. Moreover, the potential of targeting DYRK1B to treat T2DM and related metabolic diseases warrants intensive exploration.

Here, we demonstrate that energy depletion activates DYRK1B kinase activity, leading to the phosphorylation of FOXO1, nuclear translocation, and retention. DYRK1B and FOXO1 colocalize on enhancers or promoters of gluconeogenic genes, enhancing their transcription. Pharmacological inhibition of DYRK1B abrogated hyperglycemia in diabetic mice, providing a new therapeutic target for treating T2DM.

## Materials and methods

### Cell culture and treatment

HEK293 and human normal hepatocyte L02 were purchased from the Type Culture Collection of the Chinese Academy of Sciences, Shanghai, China. Mycoplasma contamination was not detected in these cell lines. All cell lines were maintained at 37°C under 5% CO_2_. All cell lines were cultured in Dulbecco’s modified Eagle’s medium (DMEM) supplemented with 10% fetal bovine serum (FBS; Gibco, 10099) and 1% PS (penicillin–streptomycin; Gibco, 15070063). Primary mouse hepatocytes were cultured in collagen I pre-treated six-well plates in medium 199 supplemented with 5% FBS and 1% PS. For transfection, HEK293 was transfected with the indicated plasmids by polyethyleneimine (Sigma), and L02 was transfected by Lipofectamine 3000 (Invitrogen, L3000015). For cell treatment, HEK293 and L02 cells were starved in serum-free DMEM supplemented with 1% fatty acid-free bovine serum albumin (BSA; Sigma, A1933-25G) for 12 h, and primary mouse hepatocytes were starved in serum-free medium 199 (Gibco, 11150059) supplemented with 1% fatty acid-free BSA for 12 h. FSK (Forskolin, MCE, HY15371) + DEX (10 μM + 2 μM for L02 cells) and cyclic adenosine monophosphate (cAMP) + DEX (100 μM + 1 μM for primary mouse hepatocytes) were added, supplemented with glucose-free DMEM (pH 7.4) without phenol red (Invitrogen, A14430-01), supplemented with 20 mM sodium lactate, 2 mM sodium pyruvate, 2 mM l-glutamine, and 15 mM HEPES for 7 h.

### CRISPR/Cas9-mediated knockout cells

*DYRK1B* knockout (KO) cell line was constructed following the protocol from Dr Feng Zhang’s Lab [28]. Briefly, control gRNA (guide RNA) and two sgRNAs (single guide RNAs) targeting *DYRK1B* [24] were cloned into Lenti-CRISPR version 2 vector (Addgene, plasmid 52961). L02 cells were transduced with lentivirus supplemented with 1 μg/ml polybrene (Sigma). Cells were selected for 72 h, before being seeded in 15-cm plates at very low density. Clones were picked after 2 weeks, grown in 6-cm plates, and analyzed by western blotting (WB) and sequencing.

### Protein samples’ preparation and WB

For WB, whole cell or tissue extracts were prepared. For cultured cells, collected cells were washed twice with ice-cold phosphate-buffered saline (PBS) and lysed in RIPA lysis buffer [50 mM Tris (pH 7.4), 150 mM NaCl, 1% Triton X-100, 0.25% sodium deoxycholate, 1 mM ethylenediaminetetraacetic acid] with freshly made protease inhibitor (Roche, 04693159001) and phosphatase inhibitor (Roche, 04906837001) for 15 min on ice. The lysates were then cleared by centrifugation at 12 000 rpm for 10 min at 4°C, and protein concentration was measured using a BCA kit (Beyotime P0011). For tissue samples, ∼15–20 mg liver tissues were lysed in RIPA lysis buffer with freshly made protease inhibitor and phosphatase inhibitor for 10 min on ice. The lysates were then grinded using a pre-cooled grinding machine (Eppendorf) for 1 min with 5 s on and 10 s off, followed by transfer into new microcentrifugation tubes. The lysates were then sonicated using a Sonicator (SONICS VCX150) for 3 min with 5 s on and 10 s off and collected by centrifugation at 12 000 rpm for 30 min at 4°C. Protein samples were separated using sodium dodecyl sulfate–polyacrylamide gel electrophoresis (SDS–PAGE) gels and then transferred to NC membranes (Millipore, HATF00010). Membranes were blocked with 5% skim milk dissolved in TBST (Tris-buffered saline with 0.1% Tween 20 detergent) for 2 h at room temperature (RT) and incubated with primary antibodies diluted in 3% BSA overnight at 4°C. Secondary antibodies’ incubation was performed after three washes with TBST at RT. After another three washes, the blots were visualized using enhanced chemiluminescence reagents (Millipore, P90720) using the ChemiDocXRS+ System (Bio-Rad). Tubulin or GAPDH was used as a loading control for normal WB, and PARP was used as a loading control for nuclear protein extractions.

### Plasmid construction

Human DYRK1B cDNA (23761) and FoxO1 cDNA (153141) were obtained from Addgene and cloned into pcDNA5/FRT-TO vector (Invitrogen) with the indicated tag. A KOD-Plus-Mutagenesis Kit (Toyobo, SMK-101) was used to generate DYRK1B and FOXO1 site mutants as well as DYRK1B deletion truncates. The corresponding wild-type plasmid served as the amplification template. The promoter reporter plasmids were cloned into pGL4-basic vector (Promega). All constructs were confirmed by DNA sequencing. Polymerase chain reaction (PCR) primers for plasmid generation are listed in Supplementary Table S1.

### Glucose production assays

Primary mouse hepatocytes were isolated and seeded in collagen-conjugated six-well plates in medium 199 with 10% FBS and 1% PS. Briefly, 6–10 h after plating, hepatocytes were transduced with the indicated adenovirus (Ad) for 12 h and then starved in DMEM without FBS and glucose for another 12 h. After starvation, hepatocytes were washed twice with 2 ml/well warm (37°C) PBS and then cultured in 1 ml/well glucose production medium consisting of glucose-free, phenol red-free DMEM (Gibco, A2493901) containing 20 mM sodium lactate (Sigma, 1614308), 2 mM sodium pyruvate (Gibco, 11360070), 2 mM l-glutamine (Gibco, A2916801), 15 mM HEPES (Gibco, 15630130), and 1% fatty acid-free BSA (Sigma, 82002). The hepatocytes were then incubated in glucose production buffer with or without 100 μM cAMP + 1 μM DEX at 37°C for 6 h. A total of 0.2 ml culture medium was transferred to microcentrifuge tubes and cleared by centrifugation at 12 000 rpm for 5 min at 4°C. The glucose concentration was then measured with a colorimetric Glucose Assay Kit (BioAssay, EBGL005). Collected hepatocytes were washed twice with ice-cold PBS and lysed in RIPA lysis buffer with fresh protease inhibitor for 10 min on ice. Lysates were cleared by centrifugation at 12 000 rpm for 10 min at 4°C, and protein concentration was measured using the BCA kit (Beyotime, P0009). The glucose production was normalized to protein concentration. The results of HGP were then normalized to the control group without cAMP and DEX treatment.

### Real-time qPCR

Total RNA was prepared using the RNeasy Plus Universal Mini Kit (Qiagen, 73404) and then reverse-transcribed into cDNA using the High-Capacity Reverse Transcription Kit (Thermo Fisher, 4374967) according to the manufacturer’s instructions. The messenger RNA (mRNA) levels were quantified and detected with SYBR Green Realtime PCR Master Mix (Toyobo, QPK-201) on the LightCycler 480 instrument II (Roche Life Science, France). Housekeeping gene, the 36B4, was used to normalize RNA in RT-qPCR (quantitative reverse transcription PCR) samples.

For cells, total RNA from fresh cells was extracted using the TRIzol (Invitrogen, 15596018) following the manufacturer’s instructions. A total of 1 μg RNA was used to reverse transcribe RNA using the High-Capacity Reverse Transcription Kit. GAPDH was used to normalize RNA in RT-qPCR samples.

### RNA-seq

Primary hepatocytes were treated with vehicle (DMSO) in fed state (fed_vehicle), with vehicle (DMSO) in fasted state (fasted_vehicle), or with AZ191 in fasted state (fasted_AZ191). Total RNA was extracted using the RNeasy Mini Plus Kit (Qiagen), and 1.3 μg of this RNA was utilized for constructing sequencing libraries using the VAHTS Universal V8 RNA-seq Library Prep Kit for Illumina (Vazyme). Each library was tagged with unique barcodes, pooled according to their quantities, and subsequently sequenced on an Illumina NovaSeq 6000 platform (Illumina, San Diego, CA, USA).

For quality control, we employed RSeQC software (version 2.6.4) to process the RNA-sequencing (RNA-seq) data. Then, the cleaned reads were aligned to the mm10 reference genome using HISAT2 (version 2.0.5). Gene-level read counts were quantified using HTSeq-count, and differential expression analysis, including normalization, was performed using the DESeq2 package in R (version 4.0.3). Differentially expressed genes (DEGs) were defined based on specific threshold criteria: baseMean ≥ 50, |log_2_ fold change| ≥ 0.58, and FDR (false discovery rate) < 0.05.

### Dual-luciferase reporter assay

L02 cells were seeded in 24-well plates for 6 h. Firefly luciferase reporter plasmids encoding the indicated promoter region of genes (200 ng/well), the Renilla luciferase reporter plasmid (50 ng/well), and experimental plasmids were transfected. Cells were harvested 36 h after transfection as per the instructions of the Dual-Luciferase Reporter Assay kit (Promega, TM040).

### Primary mouse hepatocytes’ preparation

All primary mouse hepatocytes used in this study were isolated from 6- to 8-week-old mice following the protocol as previously described [29]. Cells were seeded in collagen I-conjugated six-well plates (2.5 × 10^6^ cells/well) and cultured in medium 199 with 10% FBS. After cell attachment, hepatocytes were transduced with Ad in appropriate multiplicity of infection (MOI) (MOI = 10 for overexpression Ad, MOI = 5 for short hairpin RNA (shRNA Ad) for 12 h, and then hepatocytes were starved for another 12 h following chemical treatments.

### Immunofluorescence

Primary mouse hepatocytes were seeded and cultured on cover slips in 24-well plates and serum-starved for 12 h, followed by cAMP + DEX treatment for 6 h. The cells were then gently washed twice in PBS and fixed in 4% paraformaldehyde (Thermo Fisher, 28908) for 15 min at RT. The fixation solution was removed, and the cells were washed in PBS two more times. Permeabilization buffer (PBS containing 0.3% Triton X-100) was added, and the cells were incubated for 30 min at RT. Hepatocytes were then incubated in blocking buffer (2% BSA in PBS) for another 30 min at RT. Primary antibodies were diluted in 3% BSA in PBS. For DYRK1B and FOXO1 localization, primary hepatocytes were stained with a mouse anti-DYRK1B (Santa Cruz, sc-390417, 1:100 dilution) and a rabbit anti-FOXO1 (CST, 2880S, 1:100 dilution) overnight at 4°C. Hepatocytes were then incubated with a corresponding anti-rabbit Alexa Flour Plus 488 (Invitrogen, A32731TR, 1:1000 dilution) and anti-mouse Alexa Flour Plus 594 (Invitrogen, A32724, 1:1000 dilution) for 1 h at RT after three washes with PBS. Cells were then incubated with DAPI for 2 min at RT for nuclear staining. After that, cells were washed with PBS three more times. Cover slips were mounted upside down on slides with mounting medium and secured with clear nail polish. Images were taken using a confocal laser microscope (ZEISS, LSM 880 NLO). To investigate nuclear export of FOXO1 site mutants, L02 cells were overexpressed with Flag-FOXO1-6A (WT or 3A) and incubated with mouse anti-Flag (Sigma, F3165, 1:500 dilution) overnight at 4°C followed by secondary antibody Flour 488 incubation for 1 h at RT. Images were taken using confocal laser microscope.

### Immunoprecipitations

Immunoprecipitations of Flag-DYRK1B and endogenous FOXO1 were performed as previously described [30]. Briefly, L02 cells overexpressed with Flag-DYRK1B were gently washed twice and lysed in immunoprecipitation (IP) buffer containing both proteinase inhibitors and phosphatase inhibitors for 30 min on ice. Lysates were transferred to 1.5-ml microtubes and cleared by centrifugation at 15 000 × *g* for 20 min. Cleared supernatants were then transferred to a new microtube and incubated with ANTI-FLAG M2 Affinity Gel (Sigma, A2220) overnight at 4°C with gentle rotation. Beads were then washed three times with wash buffer and eluted using SDS loading buffer.

To investigate DYRK1B autophosphorylation state, serum-treated and serum-starved mouse primary hepatocytes (MPHs) were harvested and incubated with Phospho-Tyrosine Mouse mAb (Sepharose Bead Conjugate, CST, 9419, 1:20 dilution) overnight at 4°C and eluted with SDS loading buffer after three times washing.

### Nuclear and cytoplasmic protein isolation

For nuclear and cytoplasmic protein preparation, fresh primary hepatocytes or L02 cells were extracted using Nuclear and Cytoplasmic Extraction Reagents (Thermo Fisher, 78833) following the manual. For DYRK1B-mediated FOXO1 site-mutants’ localization, L02 cells were transfected with Flag-FOXO1-6A (WT or 3A) and then isolated nuclear and cytoplasmic protein using the kit.

### GTT, PTT, and ITT assays

Glucose tolerance test (GTT), pyruvate tolerance test (PTT), and insulin tolerance test (ITT) assays were performed following published protocols [31]. For GTT, mice were fasted for 12 h, then 20% (w/v) glucose was intraperitoneally (i.p.) injected at 2 mg per gram of body weight. Blood glucose level was tested at 0, 15, 30, 60, 90, and 120 min after glucose injection. For PTT, mice were fasted for 18 h before receiving i.p. injection of 25% (w/v) sodium pyruvate solution at 2.5 mg per gram of body weight, then blood glucose was tested at 0, 15, 30, 60, 90, and 120 min after pyruvate injection. For ITT, mice were fasted for 8 h, then 0.1 U/ml insulin solution (Gibco) was i.p. injected at 0.001 U per gram of body weight. Then, blood glucose was tested at 0, 15, 30, 60, 90, and 120 min after insulin injection.

### Mass spectrometry

Primary hepatocytes extracted from *Dyrk1b* HepKO mice and Flox controls were transfected with Ad-encoding Flag-FoxO1, and affinity purified. Flag-FoxO1 band was then excised from the Coomassie-stained gel and analyzed by mass spectrometry.

### Cleavage under targets and tagmentation

Cleavage under targets and tagmentation (CUT&Tag) for primary hepatocytes was performed using the Hyperactive Universal CUT&Tag Assay Kit for Illumina (Vazyme, TD903) following their protocol. Briefly, fresh primary hepatocytes transduced with Ad-encoding control or 3×F-DYRK1B were harvested and counted at RT. Nuclei extracted from about 100 000 primary hepatocytes were used per sample. Nuclei were incubated with 10 μl activated ConA beads and mixed lightly, after which 0.5 μl of Flag primary antibody (CST, 14793) was added to each sample and incubated for 2 h at RT. Secondary antibodies were prepared as 1:50 dilutions in Dig-wash buffer and added to each sample after discarding primary antibodies. Cells were washed three times with Dig-wash and then incubated with pA/G-Tnp adapter in Dig-300 buffer (1:200) at RT for 1 h. Cells were washed three times for 5 min in Dig-300 buffer, resuspended in 40 μl Dig-300 buffer with 10 μl 5× TTBL, and incubated at 37°C for 1 h. Briefly, 5 μl proteinase K, 100 μl buffer L/B, and 20 μl DNA extract beads were added to the fragmented sample, vortexed well and incubated for 10 min at 55°C. Cells were washed three times with 200 μl buffer WA (1×) and 200 μl buffer WB (2×). Then DNA was eluted with 22 μl sterilized ultrapure water for 5 min at RT. Eluted DNA was amplified by PCR and purified as described and then sent for sequencing.

Nucleotide sequences in FASTQ format were processed to trim adaptors using Trim Galore (version 0.6.6, https://www.bioinformatics.babraham.ac.uk/projects/trim_galore/). The trimmed sequencing reads were aligned to the mouse genome hg38 using Bowtie2 (version 2.4.2) with default settings. Signal tracks for each sample were generated using the MACS2 [32].

### Antibodies

The information of antibodies used in this article was listed in Supplementary Table S2.

### Mice genotyping

Genotyping of wild-type and conditional hepatic deletion of *Dyrk1b* genes was performed using standard PCR system. The primers used in genotyping were listed in Supplementary Table S1.

### Mice experiments

Mice were housed in individually ventilated cages at 22°C and 50% humidity under specific pathogen-free conditions with a 12-h light–dark cycle and free access to water and diet. At weaning, age-matched male mice were grouped by cage according to genotype. For the study of metabolic dysfunction induced by high-fat diet (HFD), male mice (6-week-old) were fed HFD (in which 60% of kilocalories come from fat) and compared with Chow Diet (CD)-fed animals. The average daily food intake was computed by the amount of food supply minus the amount of unconsumed food.

Dyrk1b HepKO mice were generated by Cyagen, Guangzhou, China. Briefly, Dyrk1b exon 3 and 5 were selected for generating conditional KO. Two sgRNAs (gRNA1: GGGCATGAGAGAGATGTCGAGGG and gRNA2: TGTTTTGACGCTTTATTAGGAGG) targeting the region, Cas9, and vector were co-injected into fertilized eggs for HepKO mouse production. Founder mouse was then crossed with a C57BL/6J mouse to generate Dyrk1b-flox mice. These mice were crossed with Alb-Cre transgenic mice to produce HepKO mice, and Dyrk1b-flox (Flox) mice were used as controls.

All db/db and ob/ob mice and matched lean littermate controls (db/WT, ob/WT) were obtained from Shanghai Model Organisms Center (SMOC, Shanghai, China) and bred in-house. Wild-type C57BL/6J mice were from SMOC and bred in-house.

All animal experimental procedures were approved by the Medical Ethics Committee of Shanghai Jiao Tong University School of Medicine and were performed in accordance with the Guidelines for the Care and Use of Laboratory Animals.

### Statistical analysis

For primary hepatocytes and cell line data, all statistical results were presented as the mean ± SD of at least three independent experiments. For animal studies, age- and weight-matched male mice were randomly assigned for each group. Detailed information like sample number for each group was clarified in the corresponding figure legends. All the analysis was performed using GraphPad Prism software. The significance of differences between the experimental group and the control group was analyzed using two-tailed Student’s *t*-test: ns (*P* > .05); *P < .05; **P < .01; ***P < .001.

## Results

### Expression and functions of *Dyrk1b* in HGP

**Figure 1.**
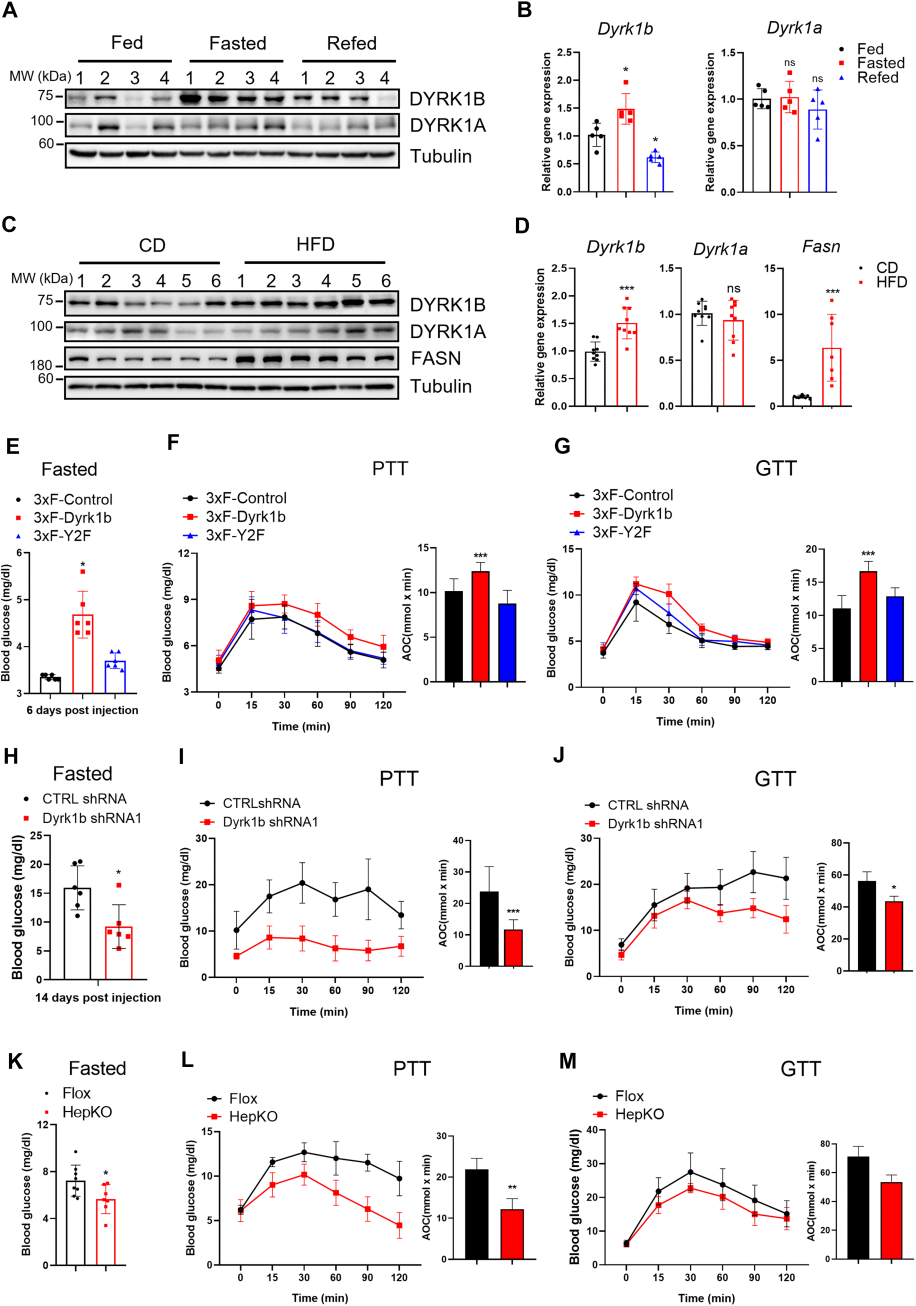
Expression and functions of Dyrk1b in HGP. (A, B) WB and RT-qPCR analysis of Dyrk1b and Dyrk1a levels in liver tissue lysates from mice under ad libitum fed, fasted overnight, and refed 4 h conditions. (**A**) WB analysis of hepatic Dyrk1b and Dyrk1a protein levels. Tubulin was used as loading control in all western samples. (**B**) RT-qPCR analysis of hepatic Dyrk1b and Dyrk1a mRNA levels. 36B4 mRNA was used to normalize RNA in RT-qPCR samples. (**C**, **D**) WB and RT-qPCR analysis of hepatic Dyrk1b and Dyr1ka in HFD-induced diabetic mice; wild-type mice fed with CD served as control. (**E**) Wild-type C57BL/6 mice at the age of six weeks were injected with Ad-encoding 3×Flag-tagged Dyrk1b wild-type (3×F-Dyrk1b) and its kinase dead mutant (Y271/273F, 3×F-Y2F). Fasted blood glucose was measured 6 days post-injection. (**F**) PTT (left) and the corresponding area of the curve (AOC) analysis (right) of mice injected with Ad-overexpressing Dyrk1b constructs. (**G**) GTT and AOC analysis of mice injected with Ad-overexpressing Dyrk1b constructs. (**H**) Diabetic db/db.BKS mice were injected with Ad encoding either scrambled control or Dyrk1b shRNA-expressing Ad at the age of 6 weeks. Fasted blood glucose was measured 6 days post-injection. PTT (**I**) and its AOC analysis and GTT (**J**) and its AOC analysis of shRNA-injected db/db.BKS mice. (**K**) Fasted blood glucose in male HFD-fed Dyrk1b HepKO and Flox control mice at week 12. (**L**) PTT glucose levels in male HFD-fed Flox control and Dyrk1b HepKO mice at week 13 and its AOC analysis. (**M**) GTT glucose levels in male HFD-fed Flox control and Dyrk1b HepKO mice at week 14 and its AOC analysis. Measurements of mice body weight, food intake, ITT, and serum insulin are provided in Supplementary Fig. S1L and M. For all graphs, data represent the mean ± SD, and *P*-values are from two-sided paired *t*-tests (*P < .05; **P< .01; ***P< .001). For panel (A), *n* = 4 mice per group. For panel (B), *n* = 5 mice per group. For panels (C), (E), (G), (H), (J), and (L), *n* = 6 mice per group. For panel (D), *n* = 9 mice per group. For panel (F), *n* = 9 mice per group. For panel (I), *n* = 5 for CTRL shRNA and *n* = 8 for Dyrk1b shRNA. For panels (K) and (M), *n* = 8 mice for Flox control and *n* = 7 for Dyrk1b HepKO.

To investigate the function of DYRK1B in metabolism, we examined the expression of hepatic *Dyrk1b* and its paralog, *Dyrk1a*, in mice under different feeding conditions. In contrast to *Dyrk1a*, the protein and mRNA levels of *Dyrk1b* in the liver increased during a 6-h fasting period and decreased upon refeeding, suggesting that it may be involved in hepatic gluconeogenesis (Fig. 1A and B). We next examined the expression of hepatic *Dyrk1b* and *Dyrk1a* in diabetic mouse models, including HFD-induced obese mice—db/db and ob/ob mice [9, 33]. Examination of protein and mRNA expression showed that hepatic *Dyrk1b* was significantly elevated in HFD-induced obese mice (Fig. 1C and D)—db/db and ob/ob mice—compared with their corresponding control mice (Supplementary Fig. S1A–D). This aligns with a recent report indicating that DYRK1B is involved in lipogenesis [22].

In the fasting state, hepatic gluconeogenesis is activated to maintain normal circulating glucose levels, but in diabetic patients, abnormal activation of gluconeogenesis is one of the most important causes of hyperglycemia [11, 34]. Therefore, we investigated if DYRK1B and its kinase activity are involved in hepatic gluconeogenesis. Ad was used in overexpression and knockdown experiments as it predominantly targets hepatocytes in mice [35]. Ad-encoding 3×Flag-Dyrk1b (3×F-Dyrk1b) and its kinase-dead mutant Y271/273F (3×F-Y2F) were administered into C57/BL6 mice through tail-vein injection, and the expression in MPHs was confirmed by western blot (Supplementary Fig. S1E) [18]. We first analyzed the blood glucose level after overexpression of wild-type DYRK1B in fasting state and observed a substantial increase in glucose levels, suggesting a role for DYRK1B in glucose metabolism (Fig. 1E). To better characterize this response, we performed PTT and GTT. PTT assesses the ability of the liver to produce glucose from noncarbohydrate sources like pyruvate. GTT assesses glucose intolerance by monitoring blood glucose levels after a glucose injection. We observed that overexpression of DYRK1B led to increased HGP following pyruvate injection (Fig. 1F). Also, GTT exhibited glucose intolerance in mice (Fig. 1G). Further, we performed ITT, which evaluates insulin sensitivity by measuring how effectively insulin lowers blood glucose levels, and found no observable effect on blood glucose levels (Supplementary Fig. S1F). Overexpression of DYRK1B did not significantly change serum insulin levels (Supplementary Fig. S1G), suggesting that hepatic DYRK1B overexpression did not reduce insulin sensitivity. We further examined if DYRK1B is responsible for the hyperglycemia in db/db mice. These mice have a mutation in the leptin receptor gene that leads to insulin resistance and progressive hyperglycemia [33, 36]. Knockdown of *Dyrk1b* with Ad-Dyrk1b shRNA1 significantly lowered fasting blood glucose levels, reduced HGP, and improved glucose tolerance in db/db mice, indicating DYRK1B protein plays a role in glucose homeostasis (Fig. 1H–J). However, insulin sensitivity and serum insulin levels were comparable between two groups of db/db mice (Supplementary Fig. S1H and I).

To validate the results from Ad-mediated overexpression and knockdown, we ablated *Dyrk1b* in hepatocytes (HepKO) by crossing *Dyrk1b* flox/flox mice with Albumin-Cre mice (Alb-Cre) [37]. Specific ablation of *Dyrk1b* in hepatocytes without affecting its expression in other tissues was confirmed by western blots (Supplementary Fig. S1J). To investigate the impact of DYRK1B depletion in a metabolic environment characterized by enhanced gluconeogenesis, HepKO mice and control mice (*Dyrk1b* flox/flox, Flox) were fed an HFD that typically leads to abnormalities in glucose and lipid metabolism [38–40]. Body weights and food intake were comparable between the two groups of mice (Supplementary Fig. S1K and L). However, HepKO mice exhibited significantly lowered fasting blood glucose levels (Fig. 1K). Moreover, reduced HGP and increased glucose tolerance were observed in HepKO mice as shown by PTT and GTT assays, indicating the requirement of DYRK1B in promoting gluconeogenesis in the liver (Fig. 1L, M). In contrast, insulin sensitivity did not change significantly in HepKO mice, suggesting that hepatic KO of Dyrk1b did not become less responsive or resistant to insulin (Supplementary Fig. S1M). Interestingly, HepKO mice exhibited lower serum insulin levels, although the values were statistically insignificant (Supplementary Fig. S1N). This suggests that the hepatic KO of Dyrk1b improves overall glucose homeostasis. In sum, our findings demonstrated that DYRK1B plays a critical role in regulating hepatic glucose metabolism.

### DYRK1B promotes HGP by upregulating gluconeogenic enzymes

**Figure 2.**
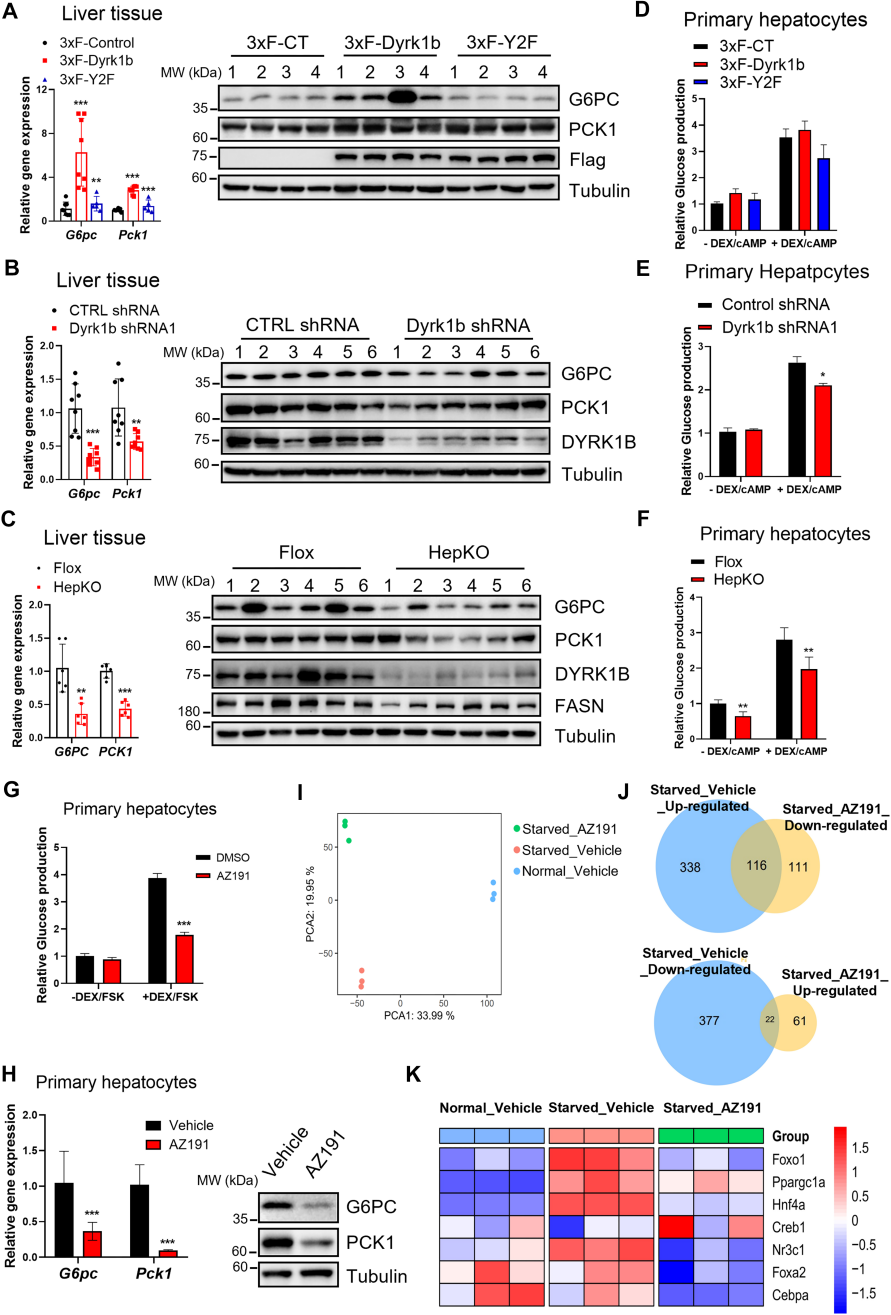
Dyrk1b promotes HGP by upregulating gluconeogenic genes. (A–C) RT-qPCR and WB analysis of gluconeogenic genes G6pc and Pck1 in liver lysates of (**A**) C57BL/6 mice overexpressing 3×F-Dyrk1b and 3×F-Y2F, (**B**) shRNA-mediated Dyrk1b knockdown in db/db.BKS mice, and (**C**) HFD-fed Dyrk1b HepKO mice. (D–F) Glucose production assay in primary hepatocytes (**D**) overexpressing 3×F-Dyrk1b and 3×F-Y2F, (**E**) shRNA-mediated Dyrk1b knockdown, and (**F**) Dyrk1b HepKO. For glucose production assay, primary hepatocytes were starved overnight followed by 10 μM FSK and 2.5 μM DEX treatment for another 6 h before glucose measurement. Glucose production(**G**) and WB and RT-qPCR analysis (**H**) in primary hepatocytes treated with or without AZ191. Primary hepatocytes were starved overnight, followed by 10 μM FSK, 2.5 μM DEX with or without 10 μM AZ191 treatment for another 6 h. Primary hepatocytes were treated with vehicle (DMSO) in fed state (fed_vehicle), with vehicle (DMSO) in fasted state (fasted_vehicle), or with AZ191 in fasted state (fasted_AZ191). (**I**) Cluster image showing global sample distribution profiles and relationships analyzed by *t*-distributed stochastic neighbor embedding based on the data from DGE (*n* = 3). (**J**) DEGs between groups were identified. The Venn diagram illustrates two clusters of DEGs: one cluster was upregulated during fasting but downregulated after AZ191 treatment, and the other was downregulated during fasting but upregulated after AZ191 treatment. There were 116 and 22 overlapping genes in these clusters, respectively. (**K**) The relative expression levels of gluconeogenesis-related transcription and co-transcription factors were depicted in the heatmap. For all graphs, data represent the mean ± SD, and *P*-values are from two-sided paired *t*-tests (*P < .05; **P< .01; ***P< .001). For panel (A), *n* = 4 mice in each group for WB and *n* = 7 mice in each group for RT-qPCR. For panel (B), *n* = 6 in each group for WB and *n* = 8 in each group for RT-qPCR. For panel (C), *n* = 6 in each group. For panels (D)–(G), data shown represent three independent experiments.

Gluconeogenesis is regulated mainly at the transcriptional level of rate-limiting enzymes, including G6pc and Pck1 (PEPCK) [11, 34]. To determine the role of Dyrk1b in the regulation of hepatic gluconeogenesis, we analyzed the expression of G6pc and Pck1 in liver tissues from mice with *Dyrk1b* overexpression, knockdown, and HepKO mice, respectively. Overexpression and shRNA experiments were performed using Ad. Overexpression of wild-type *Dyrk1b*, but not kinase-inactive mutant, led to increased mRNA levels of *G6pc* and *Pck1* and a slight increase in their protein levels (Fig. 2A). Conversely, *Dyrk1b* knockdown led to reduction in the mRNA levels of *G6pc* and *Pck1* and a slight reduction in protein levels (Fig. 2B). Further, ablation of Dyrk1b in mice livers (HepKO) resulted in significant reduction in both mRNA levels and protein levels of *G6pc* and *Pck1* (Fig. 2C). The above results suggested that DYRK1B plays a key role in regulating gluconeogenic enzymes.

To further substantiate the role of DYRK1B in hepatic gluconeogenesis, we investigated glucose production in MPHs after Ad-mediated overexpression or shRNA knockdown. Glucose production induced by DEX, a glucocorticoid analog and cAMP, was only slightly upregulated by overexpression of wild-type *Dyrk1b* (Fig. 2D) but significantly lowered with *Dyrk1b* knockdown (Fig. 2E). Additionally, glucose production in MPHs extracted from HepKO mice was significantly lower than in controls (Fig. 2F). Inhibition of DYRK1B using its specific kinase inhibitor, AZ191 [41], also decreased glucose production (Fig. 2G) as well as the expression of *G6pc* and *Pck1* (Fig. 2H) (quantification is provided in Supplementary Fig. S2A).

Gluconeogenesis is primarily regulated by two independent signaling pathways during the fasting and refeeding state. Glucagon increases gluconeogenesis by activating the cAMP pathway and CRTC2 pathways [11, 12], while circulating insulin inhibits gluconeogenesis by phosphorylating and inactivating the forkhead transcription factor FOXO1 [11, 12, 34]. To identify genes essential for gluconeogenesis, we focused on genes that are upregulated during starvation. If DYRK1B plays a role in gluconeogenesis, then blocking DYRK1B should specifically affect these genes. Therefore, we performed mRNA expression profiling by RNAseq in MPHs treated with AZ191 under normal and serum-starved conditions. DMSO was used as vehicle. Sequence reads were aligned to mm10 reference genome. DEGs were defined based on a baseMean of at least 50, a fold change of 1.5, and an FDR < 0.05. *t*-distributed stochastic neighbor embedding analysis showed a clear distinction among the three groups of samples (Fig. 2I). Serum starvation leads to increased gluconeogenesis and fatty acid oxidation to adapt to energy deprivation [34]. We observed upregulation of 454 genes and downregulation of 399 genes (Fig. 2J, blue circles). Treatment with AZ191 in the starved state resulted in significant changes in gene expression, with 83 genes upregulated and 227 genes downregulated [Fig. 2J (yellow circles) and Supplementary Fig. S2B]. To identify the genes regulated by DYRK1B during the starvation response, we compared the genes upregulated in starved hepatocytes with those downregulated in AZ191-treated starved hepatocytes and found 116 genes (Fig. 2J). Many of these genes are known regulators of autophagy and starvation. Importantly, they included several genes involved in gluconeogenesis, including Foxo1, Ppargc1a, Hnf4a, Creb1, Nr3c1, Foxa2, and Cebpa (Fig. 2K). This indicated that DYRK1B may activate hepatic gluconeogenesis via upregulation of FOXO1.

### DYRK1B colocalizes and interacts with FOXO1 during starvation

**Figure 3.**
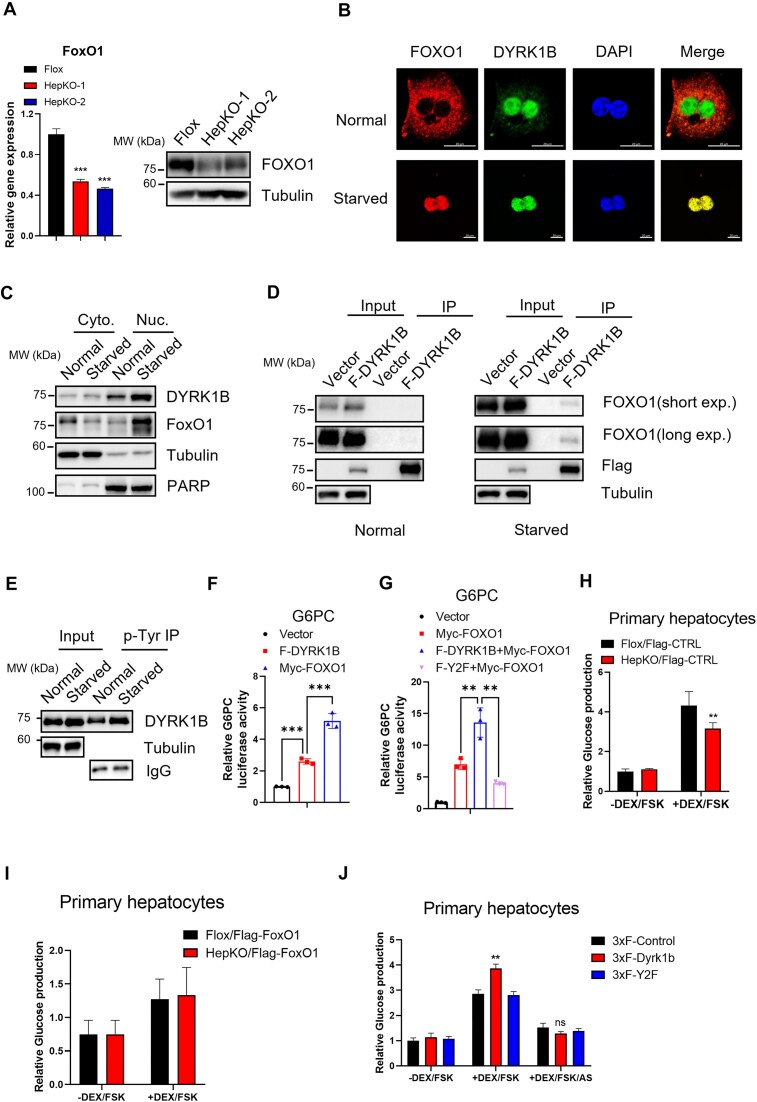
Dyrk1b colocalizes and interacts with FoxO1 during starvation. (**A**) RT-qPCR and WB analysis of FoxO1 in HepKO primary hepatocytes. RNA and protein samples were extracted from primary hepatocytes of male (HepKO-1) and female mice (HepKO-2). (**B**) Confocal images of FOXO1 and DYRK1B staining in MPHs in normal state (cultured with 10% FBS) or in serum-starved state (cultured without FBS for 12 h followed by 10 μM FSK and 2.5 μM DEX treatment for another 6 h). Nuclei were stained with DAPI. Scale bar, 20 μm. (**C**) WB analysis of endogenous DYRK1B and FOXO1 in cytoplasmic and nuclear fractions of primary hepatocytes under normal or serum-starved conditions. Tubulin was used as cytoplasmic fraction control and PARP was used as nuclear fraction control. (**D**) Co-immunoprecipitation of Flag-DYRK1B and endogenous FOXO1 in L02 cells in normal (left panel) or serum-starved states (right panel). FOXO1 immunoprecipitated by Flag-DYRK1B was analyzed by WB. (**E**) Tyrosine phosphorylation of Dyrk1b was analyzed in normal and serum-starved states as evaluated by anti-p-Tyr immunoprecipitation in primary hepatocytes. (**F**) L02 cells were transiently co-transfected with G6pc luciferase reporter, TK-Renilla reporter, and Flag-DYRK1B or Myc-FOXO1, and luciferase reporter expression was analyzed. (**G**) G6pc luciferase reporter assay was performed in L02 cells where Myc-FOXO1 was co-overexpressed with empty vector or Flag-DYRK1B (F-DYRK1B) or its kinase dead mutant (F-Y2F). (**H**) Glucose production in Flox control and Dyrk1b HepKO primary hepatocytes treated with or without 10 μm FSK and 2.5 μM DEX. (**I**) Overexpression of Flag-FoxO1 in Flox control and Dyrk1b HepKO rescued the loss of glucose production in Dyrk1b HepKO hepatocytes. (**J**) Glucose production in primary hepatocytes transduced with Ad-overexpressing F-Dyrk1b or F-Dyrk1b-Y2F, and treated with vehicle, or with 10 μm FSK and 2.5 μM DEX, or with 10 μm FSK, 2.5 μM DEX, and 10 μM AS1842856 (AS, a specific FoxO1 inhibitor). For panels (C)–(J), data shown represent three independent experiments. For panels (F) and (G), cells were serum-starved overnight. For panels (H)–(J), all primary hepatocytes were serum-starved overnight before treatment with chemicals.

RNA-seq analysis revealed that AZ191 treatment downregulates FoxO1 expression. Analysis of FOXO1 protein levels in *Dyrk1b* KO hepatocytes showed a strong reduction in both protein and RNA levels (Fig. 3A). To assess the function of DYRK1B on the transcriptional output of FoxO1-controlled promoter, we used a firefly luciferase reporter system driven by a constitutive FoxO1 promoter (Supplementary Table S1) [29]. Wild-type DYRK1B overexpression, but not kinase-dead mutant, promoted the activity of *FoxO1* promoter in L02 cells (Supplementary Fig. S3A). Also, it resulted in the upregulation of FOXO1 protein, suggesting that kinase function of DYRK1B is required for the promotion of FOXO1 expression (Supplementary Fig. S3B). Conversely, knockdown of *Dyrk1b* reduced FOXO1 protein in MPHs (Supplementary Fig. S3C). To explore the relationship and dynamics of FoxO1 and DYRK1B localization, we performed immunofluorescence (IF) experiments in normal and starved conditions. We confirmed that FOXO1 translocates to the nucleus during serum starvation, as previously reported [42], whereas DYRK1B remained in the nucleus in both normal and serum-starved states (Fig. 3B). We further verified the distribution of FOXO1 and DYRK1B by generating nuclear and cytoplasmic extracts from starved and normal cells (Fig. 3C), which supported the findings of IF experiment. We then asked if an interaction between DYRK1B and FOXO1 proteins occurs in normal or starved cells. In L02 cells overexpressing Flag-DYRK1B, FOXO1 interacted with DYRK1B during starvation but not in the normal state (Fig. 3D). Additionally, FOXO1 interacted with kinase-inactive DYRK1B Y2F, as well as the DYRK1B R102C mutant (Supplementary Fig. S3D). Interestingly, we observed that Dyrk1b expression was elevated during starvation, predominantly in the nucleus (Fig. 3C). It has been reported that DYRK1B, like other kinases in the DYRK family, undergoes autophosphorylation on a tyrosine residue in the activation loop during protein synthesis [43, 44]. We assessed the activation status of DYRK1B by immunoprecipitating tyrosine-phosphorylated proteins using a pan phospho-tyrosine antibody and probing with DYRK1B antibody. We observed a corresponding increase in phosphorylation, which paralleled the increase in DYRK1B protein levels, suggesting that newly synthesized DYRK1B is active (Fig. 3E).

We investigated whether the interaction with DYRK1B impacts the transcriptional activity of FOXO1 on gluconeogenic genes. We used a G6pc promoter-driven reporter system [29] (primer sequences in Supplementary Table S1) that is activated by FOXO1 [11, 34]. In starved condition, DYRK1B overexpression produced only a ∼2-fold increase, while FOXO1 overexpression caused a ∼5-fold increase in reporter activity (Fig. 3F). Interestingly, co-overexpression of DYRK1B with FOXO1 significantly enhanced the transcriptional activity of FOXO1 on the G6PC promoter, whereas kinase-dead mutant Y2F did not show this effect (Fig. 3G). Given that DYRK1B and FOXO1 interact under starved conditions, and their co-expression significantly enhances the FOXO1 reporter activity, it suggests that DYRK1B kinase activity promotes the transcriptional activity of FOXO1.

To confirm that the function of DYRK1B in upregulating gluconeogenesis relies on FOXO1, glucose production was examined after overexpressing FOXO1 in MPHs. Glucose production was reduced by about ∼30% in *Dyrk1b* KO MPHs (Fig. 3H). This reduction was rescued by overexpression of Flag-FOXO1 (Fig. 3I). Also, overexpression of wild-type Dyrk1b, but not kinase-inactive mutant, significantly increased glucose production (Fig. 3J). Further, treatment with AS1842856 (AS), a specific inhibitor for FOXO1 [45] attenuated the production of glucose, underscoring the central role of FOXO1 in glucose production (Fig. 3J). Thus, DYRK1B promoted glucose production in a kinase-dependent and FOXO1-dependent manner.

### DYRK1B-mediated phosphorylation is required for FOXO1 nuclear retention

**Figure 4.**
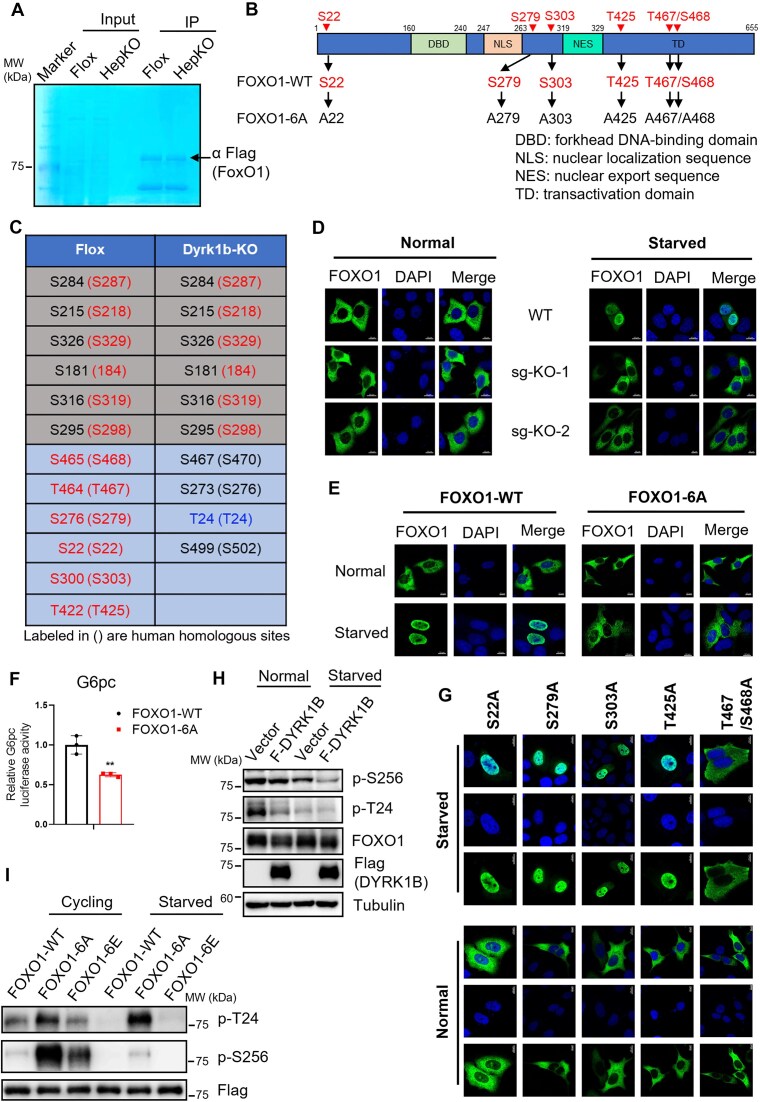
DYRK1B phosphorylates FoxO1 to promote its nuclear localization. (A–C) Flag affinity purification of Flag-FoxO1 from Flox control or Dyrk1b HepKO primary hepatocytes under serum-starvation condition. Whole cell extracts from primary hepatocytes were affinity purified with Flag beads, and eluates were analyzed by SDS–PAGE and Coomassie staining. Arrow indicates the location of FoxO1 in the Coomassie blue stained gel (**A**). (**B**, **C**) Phosphorylation sites identified by mass spectrometry analysis. Novel sites are listed in rows 7 to 12 in the Flox column (S22, S279, S303, T425, T467, and S468). (**D**) Confocal analysis of endogenous FOXO1 in Dyrk1b KO cell lines (sg-KO-1 and sg-KO-2) under fed or fasted conditions. DYRK1B sgRNA KO efficiency is provided in Supplementary Fig. S4B. (**E**) Confocal analysis of Flag-tagged FOXO1-WT or FOXO1-6A mutant in L02 cells under normal or serum-starved conditions. (**F**) G6pc luciferase reporter assay in L02 cells transfected with FOXO1-WT or FOXO1-6A. (**G**) Confocal analysis of DYRK1B-mediated phosphorylation site mutants in L02 cells under normal or serum-starved conditions. WB analysis of pFOXO1-S256 (p-S256) and pFOXO1-T24 (p-T24) in L02 cells overexpressing Flag-DYRK1B (**H**) or FOXO1-WT, FOXO1-6A, or FOXO1-6E (**I**) in normal or serum-starved conditions. For all graphs, data represent the mean ± SD, and *P*-values are from two-sided paired *t*-tests (*P < .05; **P< .01; ***P< .001). For panels (D), (E), and (G), nuclei were stained with DAPI; scale bar, 10 μm. For panels (F), (H), and (I), data shown represent three independent experiments. For panel (C), bracketed sites are corresponding homologous sites in humans. Scale bars, 10 μm (panels D, E, and G).

FoxOs are reported to be phosphorylated by several kinases [13, 15, 46–52]. For example, in liver, AKT phosphorylates FOXO1 at Thr24, Ser253, and Ser316 in response to insulin signal to promote FOXO1 nuclear exclusion [49, 53]. To investigate whether DYRK1B phosphorylates specific residues on FOXO1 under starved conditions, we overexpressed Flag-FoxO1 in *Dyrk1b* HepKO or control hepatocytes under starved conditions. We then performed affinity purification using Flag beads in the presence of proteinase and phosphatase inhibitors (Fig. 4A). Analysis by mass spectrometry identified six phosphorylation sites that were abolished on FOXO1 (S22, S279, S303, T425, T467, and S468, rows 7 to 12 in Flox column) in Dyrk1b-deficient cells but present in Flag-FOXO1 in control hepatocytes (Fig. 4B and C). Out of this, four (S22, S303, T467, and S468) phosphorylation sites are evolutionarily conserved, and two (S279 and T425) are partially conserved (Supplementary Fig. S4A). AKT-mediated phosphorylation of FOXO1 leads to its nuclear to cytoplasm translocation and transcriptional inhibition of G6PC and PCK1 [34, 49]. To determine the requirement of DYRK1B in localization of FOXO1, *Dyrk1b* ablated L02 cell lines were generated using CRISPR–Cas9 system with two sgRNAs targeting exon 3 and 7, respectively [24] (Supplementary Fig. S4B). IF studies showed that DEX/FSK-induced cytoplasmic-to-nuclear translocation of FOXO1 was almost completely inhibited in the *Dyrk1b* deletion clones (Fig. 4D). FOXO1 constructs with inactivating mutations in all six Dyrk1b-mediated phosphorylation sites (FOXO1-6A), failed to translocate to the nucleus after DEX/FSK stimulation (Fig. 4E). Additionally, nuclear and cytoplasmic fractionation showed that FOXO1-6A remained in the cytoplasm even after starvation (Supplementary Fig. S4C). Moreover, inhibition of DYRK1B by AZ191 in MPHs led to the retention of endogenous FOXO1 in the cytoplasm (Supplementary Fig. S4D). These results suggested that DYRK1B-mediated phosphorylation is necessary for FOXO1 nuclear retention. Furthermore, transient overexpression of the FOXO1-6A mutation in L02 cells carrying wild-type endogenous FOXO1 did not fully promote the transcriptional activity of G6PC promoter compared with transient overexpression of wild-type FOXO1 (Fig. 4F). To define the key phosphorylation site responsible for FOXO1 translocation, we generated single site mutants of Flag-FOXO1 and performed IF in L02 cells. Translocation of Thr467/Ser468A double mutant was strongly inhibited; however, translocation of other single site mutants was not affected (Fig. 4G). Interestingly, Thr467/Ser468 residues are located next to the LxxLL motif (462–466 aa) of FOXO1 and are evolutionarily conserved (Supplementary Fig. S4A). LxxLL motifs are present in the transactivation domains of proteins of the FOXO family and are believed to mediate the interaction of FOXO proteins with co-regulators [54]. Our above results suggested that Thr467/Ser468 are the crucial phosphorylation sites necessary for nuclear translocation of FOXO1.

Interestingly, we found FOXO1 peptides phosphorylated at Thr24 residue in affinity-purified Flag-FOXO1 from Dyrk1b KO hepatocytes (Fig. 4C, row 9 in Dyrk1b-KO column). Thr24 is a key AKT-mediated phosphorylation site critical for the nuclear exclusion of FOXO1 [49, 53, 55]. When activated by insulin and growth factors, AKT phosphorylates FOXO1 at three consensus sites (Thr24, Ser256, and Ser 319), leading to FOXO1 translocation from the nucleus to the cytoplasm [49, 53] and inhibition of FOXO1-target gene transcription, including gluconeogenic genes [11, 49, 56]. This AKT-mediated phosphorylation of FOXO1 at T24 is known to inactivate FOXO1, thereby improving hyperglycemia and insulin resistance in diabetes patients [49, 53]. Our findings align with this, as *Dyrk1b* ablation results in increased Thr24 phosphorylation, FOXO1 nuclear export, and inhibition of G6pc expression. *Dyrk1b* HepKO mice also show decreased blood glucose levels (Fig. 1K–M). The presence of Thr24 phosphorylation in the absence of DYRK1B indicates that DYRK1B indirectly influences AKT-mediated FOXO1 phosphorylation through an unknown mechanism. Further, DYRK1B overexpression in L02 cells resulted in significant reduction in T24 and S256 phosphorylation of FOXO1 (Fig. 4H). Additionally, we analyzed the phosphorylation levels of T24 and S256 residues in phospho-inactive (FOXO1-6A) and phospho-mimic (FOXO1-6E) mutations and observed an increase in FOXO-6A, but reduction in FOXO1-6E mutation (Fig. 4I). Given that these two sites are involved in AKT-mediated exclusion of FOXO1 from the nucleus, DYRK1B might also promote FOXO1 nuclear retention by inhibiting AKT-mediated phosphorylation of T24 and S256 through unknown mechanisms. Thus, DYRK1B phosphorylated and enhanced FOXO1 transactivation function during fasting while simultaneously suppressing the AKT-mediated phosphorylation of FOXO1 through unknown mechanisms.

### DYRK1B colocalizes with FOXO1 on gluconeogenic genes

To determine gluconeogenesis genes regulated by DYRK1B, we performed genome-wide location analysis of DYRK1B and FOXO1 in serum-starved MPHs using CUT&Tag analysis.

**Figure 5.**
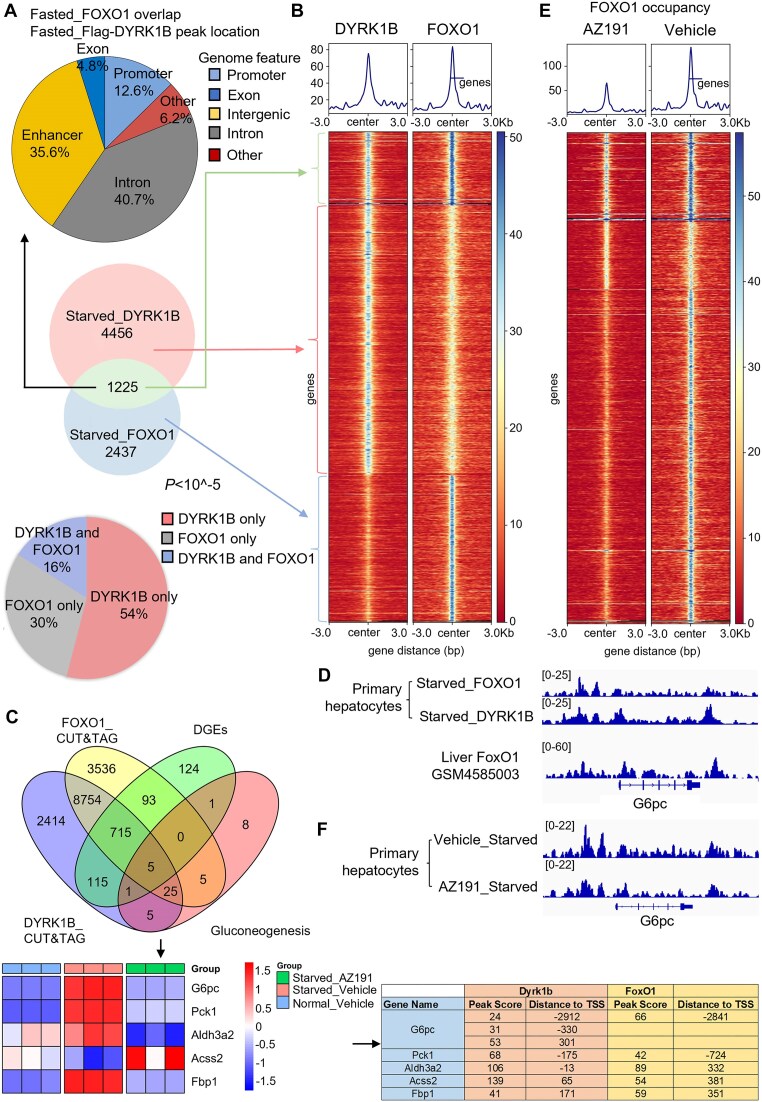
Dyrk1b colocalizes with FoxO1 on gluconeogenic genes. (**A**) Venn diagram showing the overlap between DYRK1B-binding sites and FOXO1-binding sites (circles in the middle), the genomic location of the overlapped peaks (circle at the top), and the percent distribution of peaks (DYRK1B-only, FOXO1-only, DYRK1B, and FOXO1 co-bound) (circle at the bottom). (**B**) Average signal intensities (top part) and heatmaps (lower part) of exogenously expressed Flag-DYRK1B or endogenous FOXO1 ± 3 kb from the centers of DYRK1B peaks or FOXO1 peaks. (**C**) Venn diagram showing (i) the overlap in genes that were co-occupied by DYRK1B and FOXO1 in starved condition (Fig. 5A, circles in the middle with 1225 overlapped genes) and the 116 genes that were upregulated in vehicle-treated starved condition but downregulated in AZ191-treated starved condition (Fig. 2J, upper part and (ii) these subsets of genes were then cross-checked for gluconeogenic genes. Lower part: Relative expression levels of the five overlapped genes (gluconeogenic genes that are upregulated in starved condition but downregulated by AZ191 treatment and co-occupied by DYRK1B and FOXO1) are depicted in the heatmap using *z*-score transformation method. The peak score and distance to TSS are shown in the table. (**D**) Integrative genomics viewer (IGV) image of enrichment of FOXO1 and DYRK1B at G6pc in primary hepatocytes. FOXO1 ChIP-seq data in liver tissue downloaded from GSM4585003 served as positive control. (**E**) Average signal intensities (top part) and heatmaps (lower part) of endogenous FOXO1 ± 3 kb from the centers of FOXO1 peaks in primary hepatocytes treated with or without AZ191. (**F**) IGV image of enrichment of FOXO1 at G6pc in primary hepatocytes treated with or without AZ191.

As there is no suitable antibody available for chromatin immunoprecipitation of DYRK1B for genome-wide localization analysis, we overexpressed Flag-tagged DYRK1B using Ad and performed CUT&Tag using Flag antibody. Ad-expressing Flag was used as control. To determine the loci bound by DYRK1B, signals from Flag-control were subtracted. Venn diagram analysis of the DYRK1B and FOXO1 peaks revealed 1225 overlapping peaks (Fig. 5A, middle). Chromatin localization analysis of these peaks indicated that 12.6 are localized in the promoter region. Also, a substantial number of the peaks (35.6 + 40.7) were located in the enhancer or intronic regions that harbor regulatory elements, suggesting that the genes located near these loci may be co-regulated by DYRK1B and FOXO1 (Fig. 5A, top). To further validate the extent of occupancy and potential co-regulation, we performed analysis of all the peaks occupied by DYRK1B or FOXO1 or both. As observed, we found that up to 84% peaks only had either DYRK1B occupancy (i.e. no FoxO1, 54%) or only had FOXO1 occupancy (i.e. no DYRK1B, 30%), and only about 16% peaks were co-occupied by both DYRK1B and FOXO1 (Fig. 5A, bottom). To better illustrate the chromatin occupancy and their overlap in binding sites, we generated heatmap of all the bound sites centered on the peaks. Loci with DYRK1B and FOXO1 colocalization were plotted on top (Fig. 5B, top). Motif analysis using HOMER identified motifs enriched within co-bound sites or sites bound only by DYRK1B or FOXO1 (Supplementary Fig. S5A). ClusterProfiler GO enrichment analysis of the genes in the vicinity of co-bound loci identified co-regulated genes related to metabolism, including *G6pc*, *Fbp2*, *Hdac2, and Mapk* (Supplementary Fig. S5B). To determine if DYRK1B regulates the genes co-bound by FOXO1 and DYRK1B under starved conditions, we compared them with downregulated genes following AZ191 treatment (Fig. 2J). Out of the 720 DEGs identified, 5 are involved in gluconeogenesis (Fig. 5C, lower panel). Interestingly, about 25 genes that are known to be involved in gluconeogenesis and found to be co-bound by FOXO1 and DYRK1B were not differentially expressed (Fig. 5C). Manual inspection of DYRK1B and FOXO1 peaks located near the gluconeogenic genes, including *G6pc* and *Pck1*, showed that DYRK1B and FOXO1 co-occupy their intergenic and/or promoter regions (Fig. 5D and Supplementary Fig. S5C). FOXO1 occupies the enhancer and promoter regions of *G6pc* in liver tissue (Fig. 5D, bottom track of *G6pc*, downloaded from GSM4585003 [57]). Since DYRK1B phosphorylates FOXO1 at multiple sites, including T467/S468, we asked whether DYRK1B-mediated phosphorylation affects the genomic occupancy of FOXO1. Inhibiting DYRK1B kinase activity using AZ191 in serum-starved MPHs significantly reduced the number of FOXO1 binding sites on chromatin, from 8409 peaks in the control group to 3704 peaks in treated cells (Fig. 5E). Manual inspection of peaks located at *G6pc* (Fig. 5F) and *Pck1* (Supplementary Fig. S5D) genes revealed decreased occupancy upon AZ191 treatment, indicating that DYRK1B is necessary for FOXO1 binding. Further, 836 genes downregulated in AZ191-treated cells also had DYRK1B occupancy in their promoter or enhancer regions, suggesting that these genes are direct targets of DYRK1B in hepatocytes (Supplementary Fig. S5E, upper panel). GO enrichment analysis of these 836 genes indicated involvement in metabolic processes (including lipid metabolism, sterol metabolism, and gluconeogenesis) and transcriptional regulation in the liver (Supplementary Fig. S5E, lower panel). Additionally, six gluconeogenic genes were also included in the gene list (Supplementary Fig. S5F). Thus, DYRK1B co-localized with FOXO1 in regions with specific motifs and regulated the transcriptional activity of FOXO1 on the associated genes, including gluconeogenic genes, in a kinase-dependent manner.

### Inhibition of DYRK1B by AZ191 improves glucose homeostasis in diabetic mice

**Figure 6.**
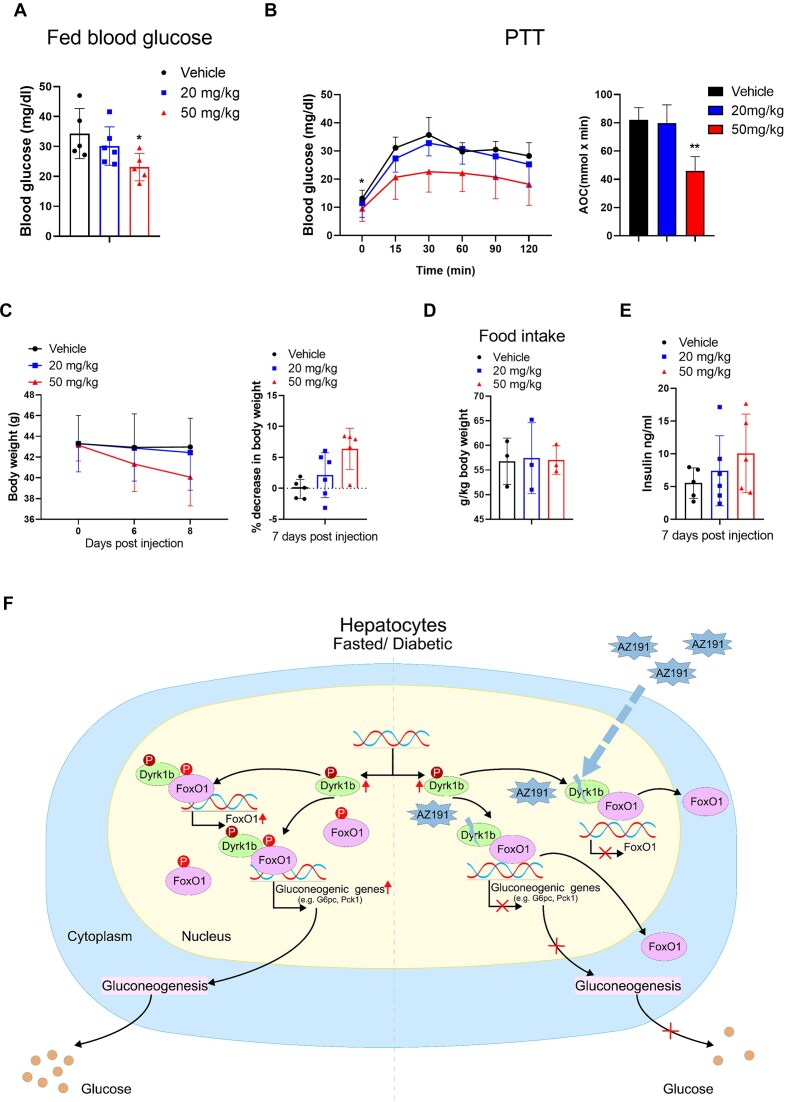
Inhibition of DYRK1B by AZ191 improves glucose homeostasis in diabetic mice. db/db.BSK mice (8-week old) were injected with either vehicle (*n* = 5) or AZ191 [with a dose of 20 (*n* = 6) or 50 (*n* = 5) mg/kg body weight, daily for 7 days, i.p.]. (**A**) Fed blood glucose was measured 5 days post-injection. (**B**) PTT (left) with its AOC analysis (right) of AZ191 injected db/db.BKS mice. (**C**) Body weight (left) and percent decrease (%) in body weight (right) of db/db.BKS mice after treatment. (**D**) The average of 2-day food intake of db/db.BKS mice during the treatment. (**E**) Serum insulin levels of these AZ191 injected db/db.BKS mice. For all graphs, data represent the mean ± SD, and *P*-values are from two-sided paired *t*-tests (*P < .05; **P< .01; ***P< .001). (**F**) Model of FOXO1 regulation by DYRK1B in diabetic or fasted state. DYRK1B resides in the nucleus, and during fasting it interacts with nuclear translocated FOXO1, and phosphorylates it to activate the transcription of gluconeogenic genes. DYRK1B also promotes the transcription of FoxO1 in this state. Inhibition of DYRK1B by AZ191 negatively regulates the expression of gluconeogenic genes.

Taking into consideration that (i) knockdown of *Dyrk1b* in diabetic mice improved blood glucose levels (Fig. 1H and I), (ii) glucose production in hepatocytes decreased after AZ191 treatment (Fig. 2G), and (iii) AZ191 treatment resulted in decreased occupancy of FOXO1 on chromatin (Fig. 5E and F), we propose AZ191 as a potential therapeutic candidate for T2DM. To test this in an *in vivo* system, db/db mice were i.p. injected with AZ191 at a dose of 20 or 50 mg/kg daily for 7 days. We observed a significant reduction in fed blood glucose levels 5 days post-injection at the dose of 50 mg/kg (Fig. 6A). In db/db mice injected with 50 mg/kg, fasting glucose levels were also lower than in controls (Fig. 6B). Glucose production response was assessed following oral (o) gavage of pyruvate after 7 days following AZ191 injection. Blood glucose levels were reduced by two times in 50 mg/kg injected mice 15 min after pyruvate challenge compared with control mice, and this induction was observed at all the time points tested (Fig. 6B). Interestingly, body weight was lower in 50 mg/kg injected mice in the absence of changes in food intake (Fig. 6C and D). This was primarily due to decreased subcutaneous adipose and visceral adipose tissue (Supplementary Fig. S6A). This result indicated that inhibition of DYRK1B also reduces lipogenesis and is consistent with our data showing that DYRK1B is involved in the regulation of lipid metabolic processes (Supplementary Fig. S5E). Notably, serum insulin levels remained unaltered (Fig. 6E), suggesting that inhibition of DYRK1B kinase activity did not affect insulin secretion or insulin sensitivity.

## Discussion

In this study, we demonstrate that Dyrk1b expression, along with its kinase activity, is significantly elevated during fasting and in diabetic mouse models. DYRK1B interacts with FOXO1 and phosphorylates it at multiple sites, importantly at residues T467/S478. This phosphorylation promotes the transcriptional activity of FOXO1. Additionally, DYRK1B-mediated phosphorylation of FOXO1 contributes to its nuclear retention and localization to chromatin. DYRK1B co-localizes with FOXO1 at enhancer or promoter regions in hepatocytes, which includes six known gluconeogenic genes, suggesting that DYRK1B promotes gene transcription by phosphorylating and modulating the function of FOXO1. Interestingly, DYRK1B also occupies genes independent of FOXO1, indicating potential novel functions related to the animal's response to fasting (Fig. 5A and B). Furthermore, inhibiting DYRK1B kinase activity using AZ191 affects FOXO1 stabilization on chromatin, improving hyperglycemia in diabetic mice, highlighting the therapeutic potential of AZ191 in diabetes treatment (Fig. 6F).

Mutation in Dyrk1b has been reported to activate adipogenic transformation and hepatic gluconeogenesis, leading to metabolic syndrome [20]. Recently, it was reported that DYRK1B promotes hepatic lipogenesis by activating mTORC2, a process that occurs in the cytoplasm [22]. However, the role of DYRK1B in the nucleus remains largely undetermined. Interestingly, in our study, we found that DYRK1B predominantly localized in the nucleus of MPHs (Fig. 3B). Therefore, we focused on the nuclear function of DYRK1B. We demonstrated that DYRK1B regulates gene transcription by interacting with and phosphorylating the transcription factor FOXO1. Our CUT&Tag studies suggest additional nuclear roles of DYRK1B, which need to be further investigated.

Hyperactivated hepatic gluconeogenesis contributes to fasting hyperglycemia in patients with T2DM [20]. FOXO1 plays a key role in insulin action by suppressing hepatic gluconeogenesis and is a potential pharmacological target for treating T2DM [10]. However, FOXO1 is a transcription factor that lacks a ligand-binding domain and is considered a poor candidate as a drug target [58]. Therefore, mechanisms regulating its activity or stability are needed. Post-transcriptional modifications of FOXO1, including phosphorylation, acetylation, and ubiquitination [10, 50, 53, 55, 59, 60], are associated with insulin resistance and glucose homeostasis. Class IIa HDAC-mediated deacetylation of FOXO1 activates FOXO1 and promotes glucose production [10]. FOXO1 is ubiquitinated and degraded by MDM2 in an AKT phosphorylation-dependent manner [60]. Our study reveals that DYRK1B phosphorylates FOXO1 and regulates gluconeogenesis. DYRK1B-mediated phosphorylation stabilizes FOXO1 on chromatin, promoting transcription of gluconeogenic genes. Inhibiting DYRK1B expression or its kinase activity could modulate glucose homeostasis. Importantly, we observed elevated expression of DYRK1B in diabetic mice, suggesting it as a potential therapeutic target for diabetes. Furthermore, our results indicate that DYRK1B binds to additional genes without FoxO1 occupancy, implying additional substrates or interaction partners of DYRK1B.

Our phospho-proteomics study indicated that DYRK1B not only phosphorylates FOXO1 at several sites (Fig. 4B), but also suppresses the phosphorylation by AKT at S256 and T24 (Fig. 4H). In the fed state, these sites are phosphorylated by a recently identified nuclear p53-AKT signalosome that mediates the nuclear export of FOXO1 and subsequent degradation by ubiquitination [61]. It would be interesting to know the molecular events initiated during fasting that lead to DYRK1B-mediated phosphorylation of FOXO1. Also, it will have important implications in T2DM. The DYRK1B inhibitor, AZ191, demonstrated a significant hypoglycemic effect in the diabetic model mouse. Both blood glucose levels and weight gain were significantly improved by AZ191 injection, suggesting that DYRK1B could be a potential drug target for the treatment of diabetes.

Previous studies have shown that inhibitors targeting both DYRK1A and DYRK1B can significantly increase human β-cell proliferation/mass and improve insulin secretion [62–67]. This effect was primarily attributed to the inhibition of their roles in cell cycle exit, thereby promoting β-cell proliferation. Our study reveals that DYRK1B plays a crucial role in gluconeogenesis by regulating the localization and transcriptional activity of FOXO1, highlighting a previously unknown function of DYRK1B. Moreover, it implies that combining inhibitors for both DYRK1A and DYRK1B could potentially offer a comprehensive approach to diabetes treatment, opening new avenues for targeted diabetes therapies that consider both β-cell proliferation and HGP.

## Supplementary Material

gkaf319_Supplemental_File

## Data Availability

DYRK1B CUT&Tag-seq data can be accessed through GEO with the accession number GSE263028; FOXO1 ChIP-seq data are available from GEO under the accession number GSM4585003; Differential Gene Expression (DGE) data are available from GSE262106. Proteomics data are available via ProteomeXchange with the identifier PXD052214.

## Notes

Present address: St Jude Children’s Research Hospital, 262 Danny Thomas Place, Memphis, TN 38105, USA
