# Supplementary material for: DYRK1B phosphorylates FOXO1 to promote hepatic gluconeogenesis
Source: Nucleic Acids Res. 2025 Apr 26;53(8):gkaf319. doi: 10.1093/nar/gkaf319 (PMC12034038; doi:10.1093/nar/gkaf319)
Supplement: gkaf319_Supplemental_File [file gkaf319_supplemental_file.docx]

**Supplementary File**

**DYRK1B phosphorylates FOXO1 to promote hepatic gluconeogenesis**

Shanshan Li^1$^, Kai Huang^2$^, Chu Xu^3$^, Hong Zhang^1^, Xiao Wang^4^, Rong Zhang^1^, Yan Lu^5, #^, Man Mohan^6 #^, Cheng Hu^1, #^

Affiliations

^1^ Shanghai Diabetes Institute, Shanghai Sixth People's Hospital Affiliated to Shanghai Jiao Tong University School of Medicine, Shanghai 200233, China.

^2^ Department of Sports Medicine, Shanghai Sixth People's Hospital Affiliated to Shanghai Jiao Tong University School of Medicine, Shanghai 200233, China.

^3^ CAS Key Laboratory of Genome Sciences and Information, Beijing Institute of Genomics, Chinese Academy of Sciences, Beijing 100101, China.

^4^ Key Laboratory of Biomedical Research Center, Sir Run Run Shaw Hospital, Zhejiang University School of Medicine, Hangzhou, Zhejiang.

^5^ Institute of Metabolism and Regenerative Medicine, Shanghai Sixth People's Hospital Affiliated to Shanghai Jiao Tong University School of Medicine, Shanghai 200233, China.

^6^ State Key Laboratory of Primate Biomedical Research, Institute of Primate Translational Medicine, Kunming University of Science and Technology, Kunming, China.

Present address: Man Mohan, St. Jude Children’s Research Hospital, 262 Danny Thomas Place, Memphis, TN 38105, USA.

^#^ The last three authors are corresponding authors

**Supplementary Figure legends:**

**Supplementary Figure S1 Dyrk1b improves glucose homeostasis.**

(A – D) WB and RT-qPCR analysis of hepatic Dyrk1b and Dyrk1a in diabetic mouse models ob/ob (A - B) and db/db.BKS (C - D) mice. FASN serve as a positive control. (E) WB analysis of Dyrk1b in MPHs infected with Ad-3xFlag-Dyrk1b (3xF-Dyrk1b) and its kinase-dead mutant (3xF-Y2F). (F– G) ITT with its AOC analysis (F) and serum insulin levels (G) of 3xF-Dyrk1b and 3xF-Y2F overexpressed mice. (H – I) ITT with its AOC analysis (H) and serum insulin levels (I) of shRNA mediated Dyrk1b knockdown mice. (J) WB analysis of Dyrk1b ablation in different tissues (liver, Quads (quadriceps femoris), SAT, BAT) and primary hepatocytes (lower panel) extracted from Flox control and Dyrk1b HepKO mice. (K- N) Body weight with its AUC analysis (K) and food intake (L) of male HFD-fed Flox control and Dyrk1b HepKO mice; ITT with its AOC analysis (M) was performed in these HFD-fed mice at week 16 and their serum insulin levels (N). For all graphs, data represents the mean ± SD, and *P* values are from two-sided paired t-tests ((**P* < 0.05, ***P*<0.01, ****P*<0.001). Data represents the mean ± SD. For A, C and F, n = 6 mice per group. For B, D and G, n = 8 mice per group. For I and J, n = 8 mice for Control shRNA and n = 7 for Dyrk1b shRNA1. For L - M, n = 8 mice per group. For N, n = 8 mice for Flox control and n = 7 for Dyrk1b HepKO. For O, n = 8 mice for Flox control and n = 6 for Dyrk1b HepKO.


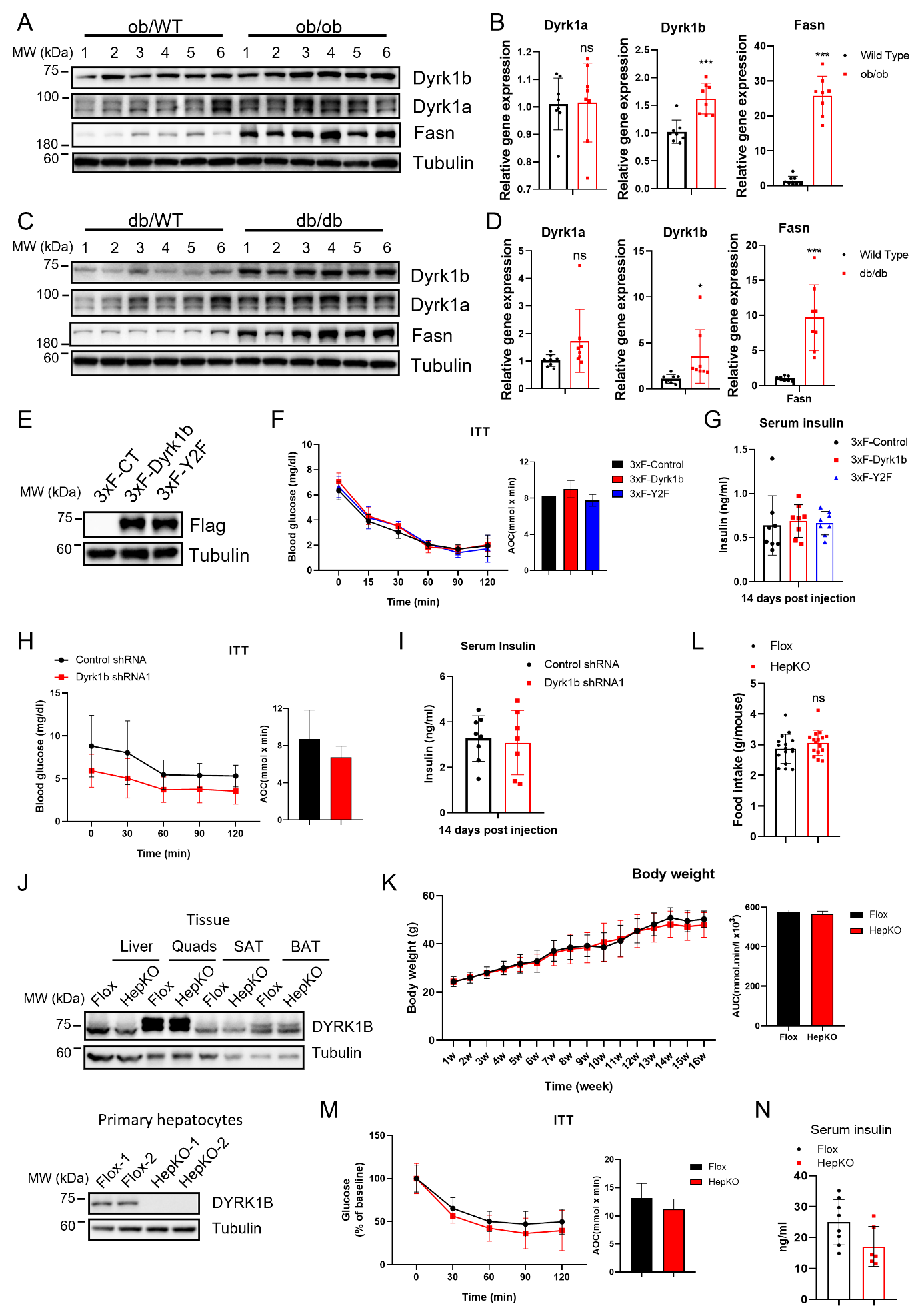


**Supplementary Figure S2 AZ191 treatment affects the expression of fasting induced genes.**

(A) Statistical analysis of Figure 2H. (B) Heatmap analysis shows that AZ191 treatment largely inhibits the expression of genes which are induced in response to fasting.


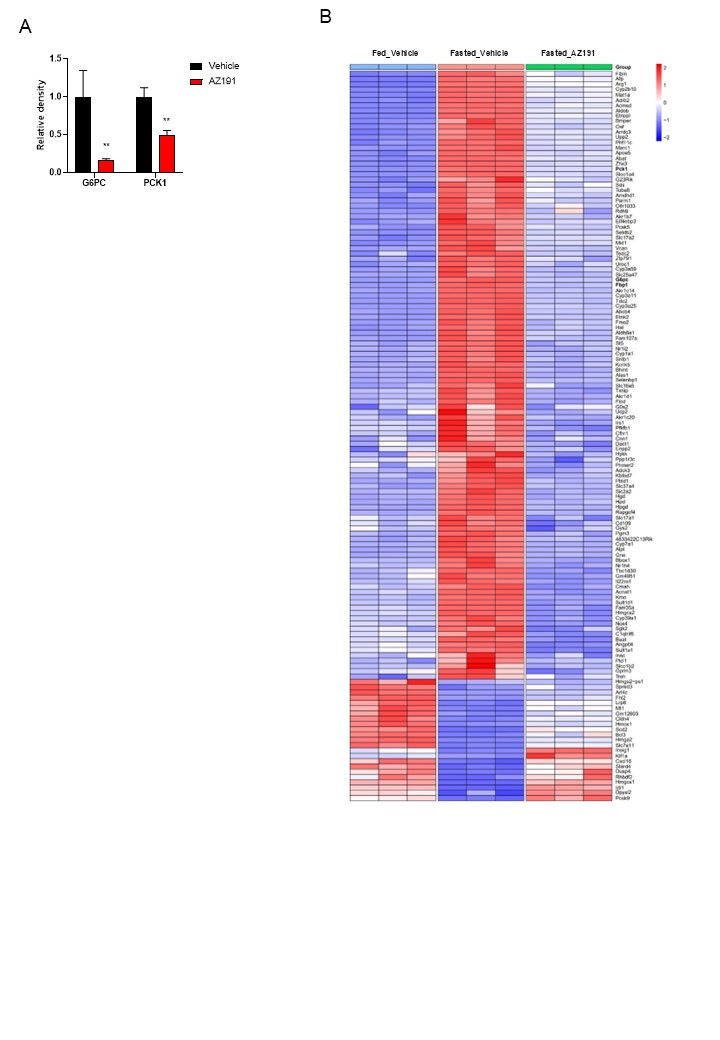


**Supplementary Figure S3 DYRK1B promotes the expression of FoxO1 in a kinase-dependent manner.**

(A) FOXO1 luciferase reporter assay was performed in L02 cells overexpressing empty vector, Flag-DYRK1B (F-DYRK1B) or kinase dead mutant (F-Y2F). FOXO1 luciferase values were normalized to the empty vector control. (B) WB (left) of FoxO1 and gluconeogenic genes in primary hepatocytes overexpressing empty vector, Flag-Dyrk1b (F-Dyrk1b) and Flag-Dyrk1b-Y2F (F-Y2F). Statistical analysis of three different experiments (right). (C) WB (left) and its statistical analysis (middle) of FoxO1 levels in shRNA mediated Dyrk1b knockdown primary hepatocytes. RT-qPCR analysis of the samples (right). (D) Immunoprecipitation of Flag-DYRK1B and its mutants (F-Y2F, F-R102C) from lysates of serum-starved L02 cells. FOXO1 immunoprecipitated by Flag-DYRK1B or its mutants were analyzed by WB. For all graphs, data shown represent three independent experiments. For C, data represents the mean ± SD, and *P* values are from two-sided paired t-tests ((**P* < 0.05, ***P*<0.01, ****P*<0.001). Data represents the mean ± SD.


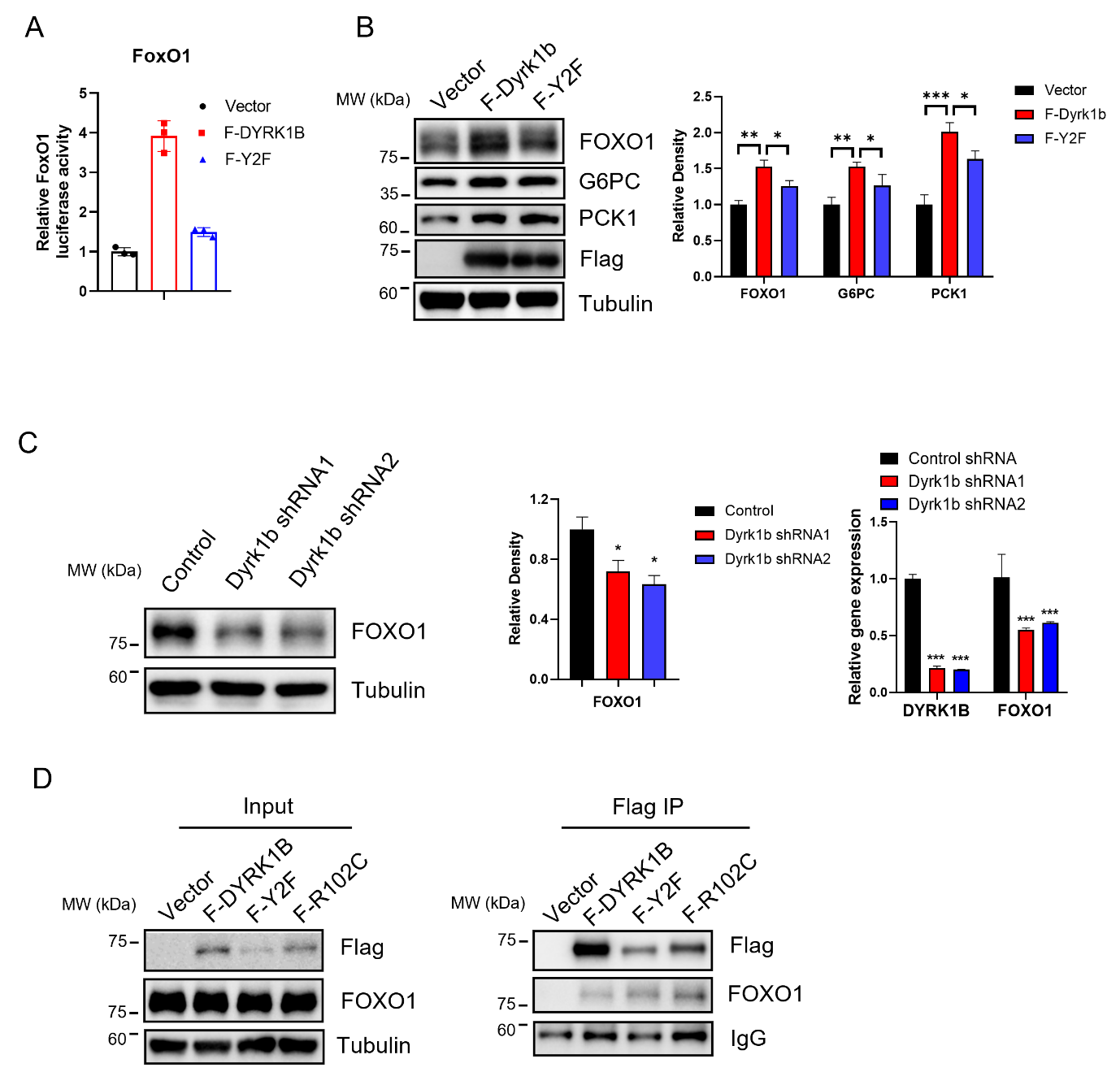


**Supplementary Figure S4 DYRK1B-mediated phosphorylation is involved in FOXO1 translocation.**

(A) Conservation analysis of DYRK1B-mediated phosphorylation sites on FOXO1, of which four are highly conserved (S22, S303, T467 and S468). (B) WB (top) and sequencing (bottom) analysis of CRISPR Cas9 generated knockout *DYRK1B* gene clones. (C) WB analysis of FOXO1-WT and FOXO1-6A cytoplasmic/nuclear distribution and their phosphorylation status of p-S256 and p-T24 in L02 cells under fed or fasted conditions. (D) WB analysis of FOXO1 cytoplasmic/nuclear distribution in starved MPHs treated with AZ191.


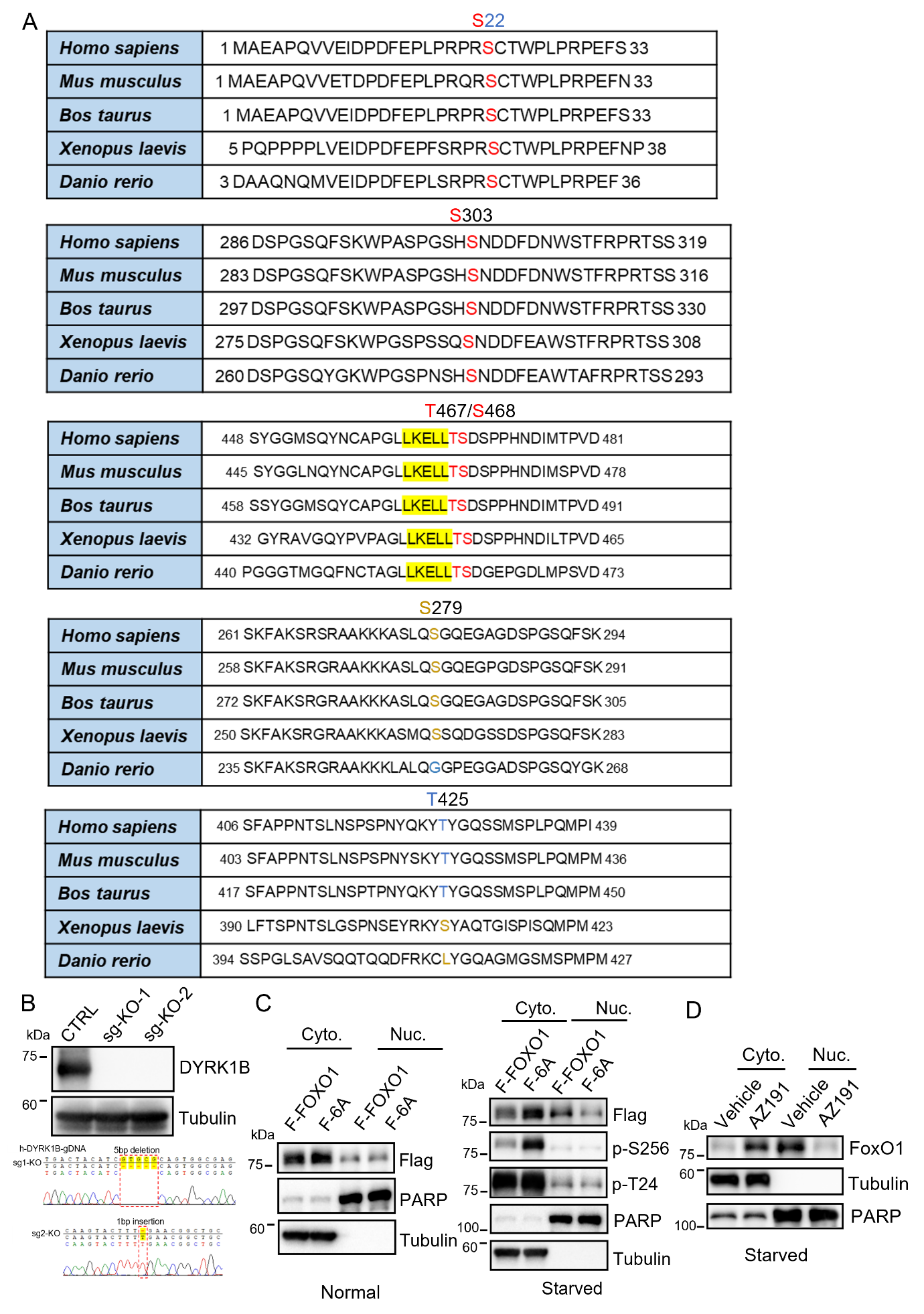


**Supplementary Figure S5 Co-occupancy analysis of DYRK1B and FOXO1 on chromatin by CUT&TAG-seq.**

(A) Significant motifs at the DYRK1B and FOXO1 co-bound peaks (left) and DYRK1B-only peaks (middle) and FOXO1-only peaks (right) in primary hepatocytes. (B) GO analysis of DYRK1B and FOXO1 co-bound genes. (C) IGV image of enrichment of FOXO1 and DYRK1B at *Pck1* in primary hepatocytes. (D) IGV image of enrichment of FOXO1 at *Pck1* in primary hepatocytes treated with or without AZ191. (E) Venn diagram showing the significant overlap between DYRK1B-binding sites and DGEs (top) followed by GO analysis of the overlapped genes (bottom). (F) Venn diagram showing the significant overlap between DYRK1B-binding sites and DGEs and gluconeogenic genes (left), the overlapped genes are shown in the table (right).


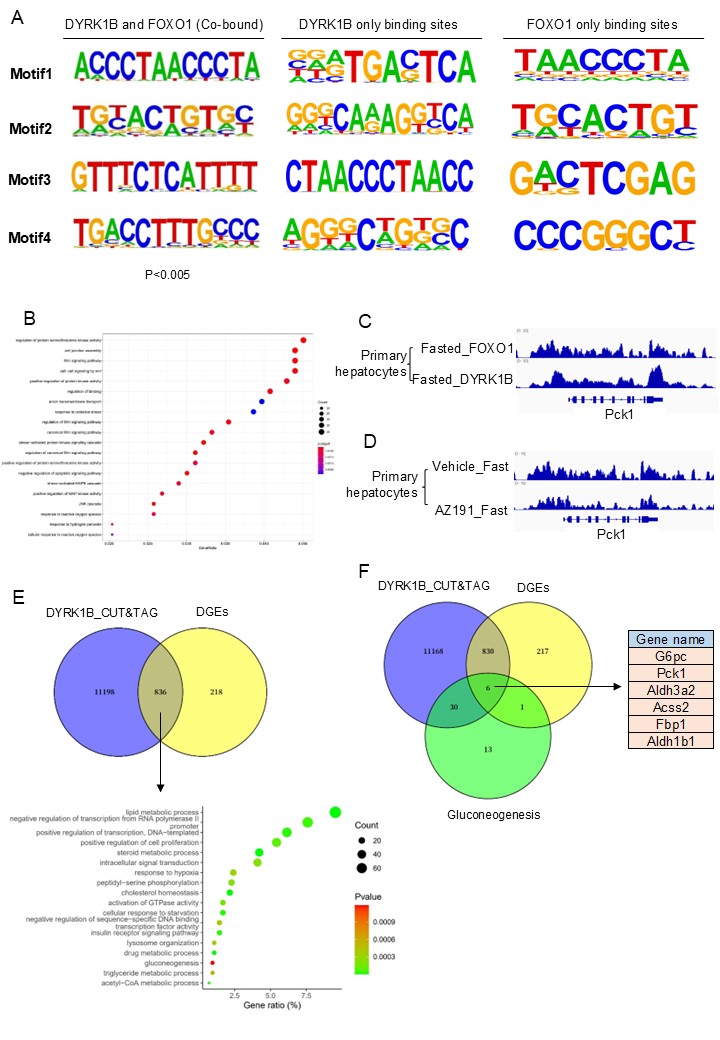


**Supplementary Figure S6 AZ191 treatment reduced the weight of adipose tissue.**

Reduction in body weight of AZ191 treated db/db.BKS mice was mainly due to decreased subcutaneous adipose (SAT) and visceral adipose (EAT). n = 6 mice for Vehicle control, n = 5 for 20mg/kg and 50mg/kg treatment group.


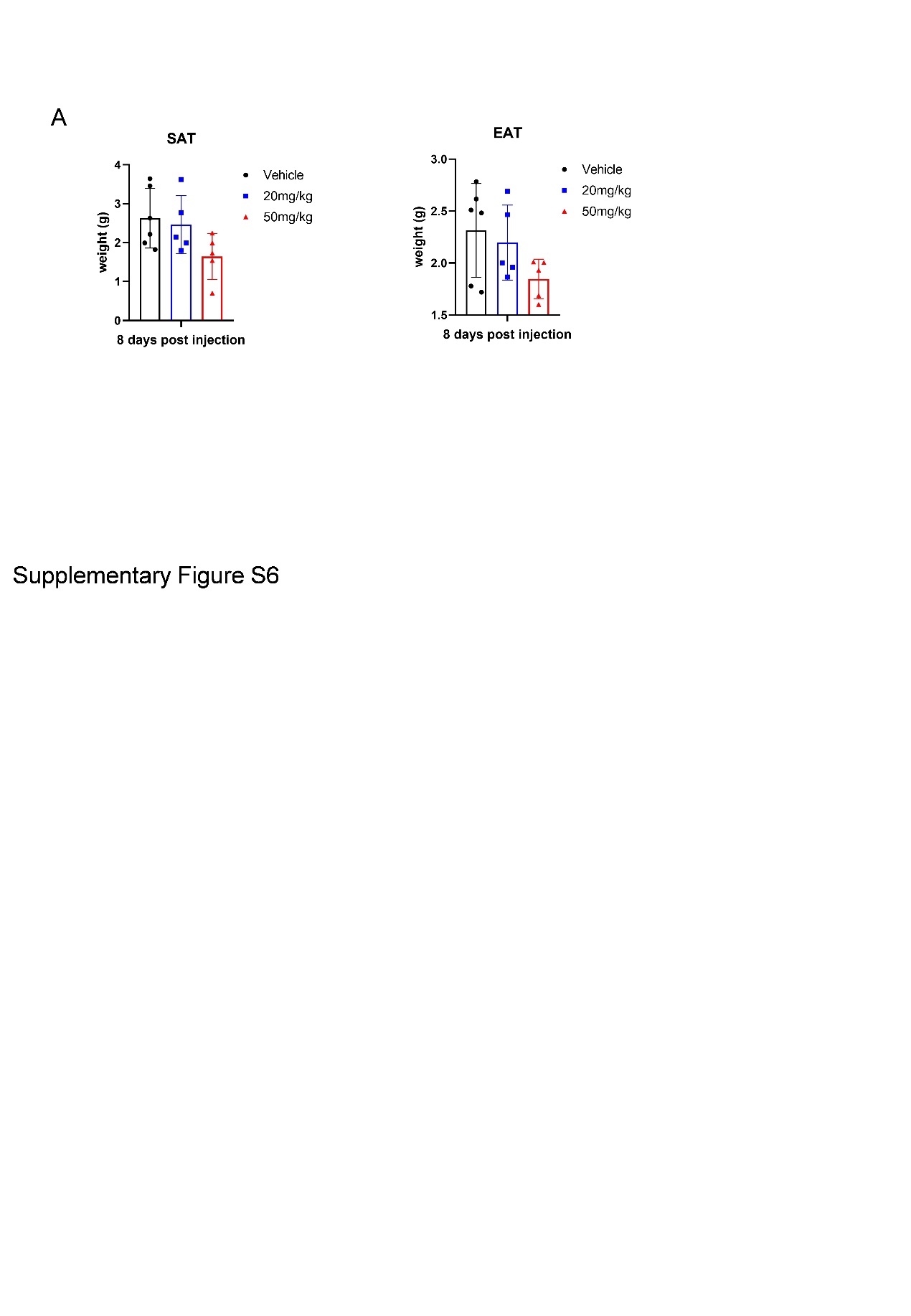


| **Supplementary Table S1** |  |
| --- | --- |
|  |  |
| **Site mutant primers** | |
| hFOXO1-S22A-R | ACGCGCGCGGCCGGGGCAGCGGCTCGAAGT |
| hFOXO1-S22A-F | CGCGCGTGCACCTGGCCGCTGCCCAGGCCGGAGTTTA |
| hFOXO1-S279A-F | CCCTCCTGGCCAGCCTGGAGAGATGCT |
| hFOXO1-S279A-R | AGCATCTCTCCAGGCTGGCCAGGAGGG |
| hFOXO1-S303A-F | GTTATCAAAGTCATCATTGGCGTGAGAGCCAGGGCTTGCA |
| hFOXO1-S303A-R | TGCAAGCCCTGGCTCTCACGCCAATGATGACTTTGATAAC |
| hFOXO1-S425A-F | CTGGATTGGCCATATGCATATTTTTGGTAGTTTGGGCTGG |
| hFOXO1-S425A-R | CCAGCCCAAACTACCAAAAATATGCATATGGCCAATCCAG |
| hFOXO1-S467/468A-F | TGGGGAGGAGAGTCAGCAGCCAGCAACTCCTTCAAG |
| hFOXO1-S467/468A-R | CTTGAAGGAGTTGCTGGCTGCTGACTCTCCTCCCCA |
| hDYRK1B-Y271/273F-F | GCGGCTCTGGATAAACTGGAAGATCCTCTGGCC |
| hDYRK1B-Y271/273F-R | GGCCAGAGGATCTTCCAGTTTATCCAGAGCCGC |
| mDYRK1B Y271/273F-F | GCGGCTCTGGATAAACTGGAAGATCCGCTGGCC |
| mDYRK1B Y271/273F-R | GGCCAGCGGATCTTCCAGTTTATCCAGAGCCGC |
| **luciferase assay reporter plasmid primers** | |
| h-G6PC-homo-F | TGGCCTCGGCGGCCAAGCTTTTGCAGGCATAGAAAATCTG |
| h-G6PC-homo-R | AGTACCGGATTGCCAAGCTTGCTATGAGTCTGTGCCTTGC |
| m-G6PC-homo-F | TGGCCTCGGCGGCCAAGCTTTTGCAGACTTCAAAAAACAG |
| m-G6PC-homo-R | AGTACCGGATTGCCAAGCTTGCTATCAGTCTGTGCCTTGC |
| h-FOXO1-homo-F | ACCTGAGCTCGCTAGCCTCGAGTACACTGGTATTATAGGATGCTT |
| h-FOXO1-homo-R | AACAGTACCGGATTGCCAAGCTTCCCGCTGACAAGGGCCG |
| **shRNA target sequence** | |
| Ad-shFoxO1-F | TGGAAACCAGCCAGCTATAAA |
| shDYRK1B-F | ccAGCATGATACAGAGATGAA |
| **Mouse genotyping primers** | |
| 1b-Cre-checking-F | GAACGCACTGATTTCGACCA |
| 1b-Cre-checking-R | GCTAACCAGCGTTTTCGTTC |
| 1b-CKO-mutant-checking-F | GTGGAGCTGTTTTGACGCTTTAT |
| 1b-CKO-mutant-checking-R | CTCTAGCGTAGATTATGGGATGACC |
| **RT-qPCR primers** | |
| mPEPCK-RT-F | CTGCATAACGGTCTGGACTTC |
| mPEPCK-RT-R | CAGCAACTGCCCGTACTCC |
| mG6PC-RT-F | GTTGAACCAGTCTCCGACCA |
| mG6PC-RT-R | CGACTCGCTATCTCCAAGTGA |
| mFoxo1-RT-F | AAGAGCGTGCCCTACTTCAA |
| mFoxo1-RT-R | TGCTGTGAAGGGACAGATTG |
| mDYRK1B-RT-F | AAGATCGTGGACTTCGGCAG |
| mDYRK1B-RT-R | TGGCCAGGTCATAGGGTGTA |
| mDYRK1A-RT-F | CCTCTGTTCAGTGGTGCCAA |
| mDYRK1A-RT-R | CTTGCTTTCGGTGCTTGGTC |
| **sgRNAs sequences** | |
| Non-targeting control sgRNA | GTATTACTGATATTGGTGGG |
| DYRK1B sgRNA-1 | CATGACTACATCGTGCGCAG |
| DYRK1B sgRNA-2 | AGGCTCGCAAGTACTTTGAA |
|  |  |

**Supplementary Table S2**

**Antibody Information**

| **Antibodies** | **Source** | **Identifier** |
| --- | --- | --- |
| DYRK1B | CST | 5672S |
| DYRK1B | Santa Cruz | sc-390417 |
| FoxO1 | CST | 2880S |
| FLAG-Tag | CST | 14793S |
| DYRK1A | Santa Cruz | sc-100376 |
| G6pc | NOVUS | NBP1-80533 |
| PCK1 | Abcam | ab70358 |
| Phospo-FoxO1(Thr24)/FoxO3a(Thr32) | CST | 9464S |
| Phospho-FoxO1(Ser256) | CST | 9461S |
| Myc-Tag | CST | 3946S |
| Phospho-Tyrosine Mouse mAb (P-Tyr-100) (Sepharose Bead Conjugate) | CST | 9419 |
| Tubulin | CST | 2146S |
| PARP | CST | 9532S |
| Fasn | CST | 3180S |
